# GroEL is an immunodominant surface-exposed antigen of *Rickettsia typhi*

**DOI:** 10.1371/journal.pone.0253084

**Published:** 2021-06-10

**Authors:** Jessica Rauch, Jessica Barton, Marcel Kwiatkowski, Malte Wunderlich, Pascal Steffen, Kristin Moderzynski, Stefanie Papp, Katharina Höhn, Hella Schwanke, Susanne Witt, Ulricke Richardt, Ute Mehlhoop, Hartmut Schlüter, Verena Pianka, Bernhard Fleischer, Dennis Tappe, Anke Osterloh

**Affiliations:** 1 Bernhard Nocht Institute for Tropical Medicine, Hamburg, Germany; 2 University Medical Center Hamburg-Eppendorf, Hamburg, Germany; Ramathibodi Hospital, Mahidol University, THAILAND

## Abstract

Rickettsioses are neglected and emerging potentially fatal febrile diseases that are caused by obligate intracellular bacteria, rickettsiae. *Rickettsia* (*R*.) *typhi* and *R*. *prowazekii* constitute the typhus group (TG) of rickettsiae and are the causative agents of endemic and epidemic typhus, respectively. We recently generated a monoclonal antibody (BNI52) against *R*. *typhi*. Characterization of BNI52 revealed that it specifically recognizes TG rickettsiae but not the members of the spotted fever group (SFG) rickettsiae. We further show that BNI52 binds to protein fragments of ±30 kDa that are exposed on the bacterial surface and also present in the periplasmic space. These protein fragments apparently derive from the cytosolic GroEL protein of *R*. *typhi* and are also recognized by antibodies in the sera from patients and infected mice. Furthermore, BNI52 opsonizes the bacteria for the uptake by antigen presenting cells (APC), indicating a contribution of GroEL-specific antibodies to protective immunity. Finally, it is interesting that the GroEL protein belongs to 32 proteins that are differentially downregulated by *R*. *typhi* after passage through immunodeficient BALB/c CB17 SCID mice. This could be a hint that the rickettsia GroEL protein may have immunomodulatory properties as shown for the homologous protein from several other bacteria, too. Overall, the results of this study provide evidence that GroEL represents an immunodominant antigen of TG rickettsiae that is recognized by the humoral immune response against these pathogens and that may be interesting as a vaccine candidate. Apart from that, the BNI52 antibody represents a new tool for specific detection of TG rickettsiae in various diagnostic and experimental setups.

## Introduction

Rickettsial infections are neglected and emerging diseases. Rickettsiae are obligate intracellular Gram-negative bacteria that are subdivided into three major pathogenic biogroups of pathogenic bacteria: the TG rickettsiae, the SFG rickettsiae and the transitional group of rickettsiae. *Rickettsia typhi (R*. *typhi) and R*. *prowazekii* constitute the TG of rickettsiae causing endemic and epidemic typhus in humans. The bacteria are transmitted to humans by arthropod vectors, causing disease that represents with high fever, headache, muscle and joint pain, nausea and vomiting. Around 50–60% of *R*. *prowazekii*-infected patients show a characteristic rash around day six after infection. In addition, neurological symptoms such as confusion and stupor are common [1]. As the bacteria infect endothelial cells of the blood vessels [2, 3] and systemically spread in the body, the infection can affect nearly all organs leading to multiorgan inflammation and dysfunction. In severe cases fatal complications such as pneumonia, encephalitis and myocarditis can occur [1, 4]. The course of disease is generally milder in *R*. *typhi* infection (<5% lethality) [5, 6] compared to the infection with *R*. *prowazekii* (20–30% lethality) [4, 6, 7]. Rickettsial infections can be treated with antibiotics, with tetracyclines being the reference agents. Appart from that, rickettsiae also respond to chloramphenicol. Despite such treatment, however, at least some rickettsial species can latently persist and cause disease years to decades after primary infection again. This is well known for *R*. *prowazekii* that can be reactivated and cause the so-called Brill-Zinser disease [8–11]. Persistence has also been demonstrated for other rickettsiae including *R*. *typhi* [12], *R*. *rickettsii* [13, 14] and *Orientia* (*O*.) *tsutsugamushi* [15], and recurrence of these bacteria cannot be excluded.

Endemic typhus caused by *R*. *typhi* is distributed worldwide, with the majority of cases occuring in coastal tropical and subtropical regions. It is highly prevalent in low-income countries in Asia [16–19] and Africa [20] and appears with steadily increasing incidence over the past twenty years also in the United States (mainly in Southern California, Texas and Hawaii [21–23]) and southern Europe (Greece, Cypres, Spain, Portugal [24–32]). Especially homeless people are at enhanced risk to acquire the infection. For example, 9.6% of the homeless were seropositive in Houston, Texas, in 2008 [33], and in France seropositivity in the homeless in Marseille dramatically increased from 0.054% (2000–2003) to 22% (2010–2013) [34]. In Europe, however, rickettsial infections are mainly diagnosed in travellers with febrile disease acquired abroad. A vaccine against rickettsial infections is still not available but highly desired.

In addition, there is a need for new techniques and methods for specific diagnosis of rickettsioses as these infectious diseases are still underdiagnosed due to the largely unspecific clinical picture and limited diagnostic tools. The gold standard is still the indirect detection of antibodies in the serum of patients by immunofluorescence test of infected cell cultures or Western blotting of bacterial lysates [35, 36].

The results of this study show that GroEL is an immunodominant surface-exposed antigen of TG rickettsiae and may represent a new vaccine candidate. In addition, antibodies against GroEL such as the recently generated murine BNI52 antibody [12], are useful new tools for various diagnostic and experimental purposes.

## Materials and methods

### Ethics statement

All animal experimentations and procedures were approved by the Public Health Authorities (Amt fuer Gesundheit und Verbraucherschutz, Hamburg; No 88/13) and performed according to the German Animal Welfare Act. Individual informed written consent has been obtained from patients as part of the diagnostic evaluation before the study.

### Patient sera

Patient sera from the German Reference Center for Tropical Pathogens at the Bernhard Nocht Institute for Tropical Medicine in Hamburg, Germany, from autochthonous and imported (travel- or migration-associated) rickettsioses cases were used. The TG rickettsiosis patient sera and their characteristics were described in a previous study [37]. The corresponding patient numbers of the sera used in this study (patient 1, 2 and 3) correspond to the patient data of patient 17, 10 and 19 of that study.

### Mice

C57BL/6, BALB/c and BALB/c CB17 SCID (CB17/lcr-Prkdc^SCID^/lcrlcoCrl) mice that lack T and B cells due to a genetic autosomal recessive mutation in the Prkdc^SCID^ allele on chromosome 16 [38] were bred and maintained in the animal facilities of the Bernhard Nocht Institute for Tropical Medicine, Hamburg. Five animals each were used for the infection with *R*. *typhi*, and five mice each were treated with PBS as a control. For experimentation the animals were housed in a biosafety level 3 (BSL3) facility. The facilities are registered by the Public Health Authorities (Behörde für Gesundheit und Verbraucherschutz, Hamburg, Germany). Mice were monitored daily with a clinical score. Animals that developed severe disease (clinical score ≥8) were euthanized immediately by inhalation of CO_2_.

### Cell lines

L929 (ATCC CCL-1) is a fibroblast cell line derived from C3H/An mice. HEK293T cells (ATCC CRL-3216) are human embryonic epithelial kidney cells. XTC-2 cells derive from the African clawed frog *Xenopus laevis* (RRID:CVCL_5610). Z232 are macrophages cells that were generated by immortalization of murine bmMΦ from C57BL/6 mice [39].

### Cultivation of rickettsiae and preparation of *R*. *typhi* lysates

Generally, all experimentations with living rickettsiae were performed under BSL3 conditions. *R*. *typhi* (strain Wilmington), *R*. *prowazekii* (strain Madrid E), *R*. *conorii* (strain Malish 7), *R*. *rickettsii* (strain Sheila Smith) and *R*. *africae* (strain ESF-5) were cultivated in L929 mouse fibroblasts. *R*. *felis* (strain Marseille-1BGXTC) was cultivated in XTC-2 cells. For all cell cultures RPMI1640 supplemented with 10% fetal calf serum (FCS), 2mM L-glutamine and 10 mM HEPES without antibiotics was used. All cell culture reagents were obtained from PAA Laboratories, Cölbe, Germany. Stocks of purified bacteria were prepared as described previously [12]. For the preparation of *R*. *typhi* lysates, bacterial stocks were inactivated by incubation at 56°C for 30 min, resuspended in lysis buffer 1 (10 mM Tris-HCl, 2 mM EDTA, 0.5% Triton X100, 0.5% NP40 and complete protease inhibitor cocktail (ThermoFisher Scientific, Darmstadt, Germany) and incubated for 30 min on ice. Lysis buffer 1 without protease inhibitors was used for proteinase K digestion. Lysis was completed by sonification in a Branson Sonifier (twenty times for 30 sec/ output control 1.5/ duty cycle 40). For 2D gel electrophoresis and mass spectrometric analyses bacteria were lyzed in lysis buffer 2 (1% sodiumdeoxycholate (SDC), 0.1 M ammonium hydrogen carbonate and complete protease inhibitor cocktail) and incubated for 5 min 98°C. Additionally, the lysates were treated with DNase (ThermoFisher Scientific, Darmstadt, Germany) for 30 min at room temperature. After centrifugation (3220xg, 10 min, 4°C) the supernatant was used.

### Determination of protein concentrations

Protein concentrations were measured by Bradford assay. A 1:2 serial dilution of BSA starting with a concentration of 1 mg/mL down to 15 μg/mL was used as standard. Bradford Reagent (ThermoFisher Scientific, Darmstadt, Germany) was added to standards and samples and the OD_630_ was determined in an MRX-II ELISA plate reader (Dynex Technologies, Germany).

### Indirect immunofluorescence test (IIFT)

Cells infected with different rickettsial species were washed three times in PBS and fixed with ice-cold acetone for 10 min at -20°C. Blocking was performed by adding 5% mouse serum (Sigma-Aldrich, Munich, Germany) in PBS for 10 min at 37°C. Then the cells were incubated with the BNI52 antibody (1.3 μg/mL) in PBS or with a mouse IgG3 isotype control antibody (1.3 μg/mL; Biolegend, San Diego, USA) for 30 min at 37°C. After washing three times in PBS, the cells were incubated with DAPI (4`,6-diamidino-2-phenylindole; 1:1000 in PBS; Sigma-Aldrich, Munich, Germany) and an anti-mouse IgG3-FITC (fluorescein isothiocyanate; 1:200; BioLegend, San Diego, USA) for 30 min at 37°C. Afterwards, cells were washed in PBS and an anti-FITC-AlexaFluor 488-conjugated antibody (1:1000 in PBS; ThermoFisher Scientific, Darmstadt, Germany) was added for 30 min at 37°C. Cells were washed again and sealed with PermaFluor (ThermoFisher Scientific, Darmstadt, Germany). Images were taken with a BZ9000 Keyence microscope (Keyence, Neu-Isenburg, Germany).

### Electron microscopy

*R*. *typhi*, *R*. *prowazekii*, *R*. *conorii* and *R*. *felis* were fixed with 4% formaldehyde in PBS at room temperature for 30–60 min followed by a second fixation step with 1% paraformaldehyde (Electron Microscopy Sciences, Hatfield, USA) and 0.025% glutaraldehyde (Electron Microscopy Sciences, Hatfield, USA) in cacodylate buffer, pH 7.2 (Electron Microscopy Sciences, Hatfield, USA). Cells were then stored and collected at 4°C. After washing with 50 mM cacodylate buffer and distilled water, samples were stained with 2% aqueous uranyl acetate (Electron Microscopy Sciences, Hatfield, USA) for 30 min at room temperature and dehydrated through a graded series of ethanol (50%, 70%, 95%, 100%). Following embedding in LR white resin (medium grade; London Resin Company, Reading, UK), samples were allowed to polymerize at 57°C in the oven. 60 nm thick sections were cut with an Ultracut UC7 microtome (Leica, Wetzlar, Germany) and collected on 300 mesh nickel grids (Plano, Wetzlar, Germany) for immunogold labeling. The BNI52 antibody was applied at the concentration of 1 μg/mL in the incubating solution consisting of 0.5% BSA (Sigma-Aldrich, Munich, Germany) in PBS (PAA Laboratories, Cölbe, Germany). The sections were incubated for 1h at room temperature, followed by 1h at 4°C in the antibody solution. After the incubation period, the sections were rinsed in PBS and further incubated with a goat-anti mouse colloidal gold-conjugated secondary antibody (1:10; 12 nm gold particles; Jackson ImmunoResearch, Cambridgeshire, UK) for 1h at room temperature. Nickel grids (Sigma-Aldrich, Munich, Germany) were rinsed in PBS and stained for 45 sec with 1% aqueous uranyl acetate. Sections were observed under a Tecnai Spirit electron microscope (FEI, Eindhoven, The Netherlands) operating at 80 kV and images were recorded with a digital CCD camera (Zeiss, Oberkochen, Germany).

### Proteinase K digestion

Individual reactions were performed with *R*. *typhi* lysate (2.5 μg protein) without protease inhibitor and 0.075 U proteinase K (Sigma Aldrich, St. Louis, USA) in reaction buffer (16.6 mM Tris-HCl, 3.3 mM CaCl, pH 8). After an incubation period of 1 min, 5 min, 10 min, 30 min, 1h or 24h at 37°C, the proteinase K was inactivated in the reactions for 5 min at 95°C and the samples were applied to Western and Dot blotting.

### Precipitation of biotinylated *R*. *typhi* surface proteins with streptavidine-coupled agarose

For the biotinylation of surface proteins, *R*. *typhi* bacteria were adjusted to a concentration of 2.5×10^8^/mL in PBS. 2.2 mg EZ-link sulfo NHS-biotin reagent (ThermoFisher Scientific, Darmstadt, Germany) was dissolved in 500 μl H_2_O and 200 μl of this stock solution was added to 1 mL *R*. *typhi* suspension. Bacteria were incubated with the biotinylation reagent for 30 min on ice. Afterwards, bacteria were washed three times with PBS/100 mM glycin, finally resuspended in 50 μl PBS and inactivated for 30 min at 56°C. 450 μl lysis buffer (150 mM NaCl, 20 mM Tris, 0.5% Triton-X 100) was added and bacteria were incubated 30 min on ice. Bacterial lysates were centrifuged 21130×g and 4°C for 20 min to eliminate cell debris. The supernatants were used for the precipitation of biotinylated proteins with streptavidin agarose (catalog no. 20349, Pierce, Germany). 100 μl of the streptavidin agarose was washed twice with three volumes of lysis buffer in Eppendorf tubes. 490 μl of the lysate of *R*. *typhi* containing the biotinylated surface proteins was added and incubated with the streptavidin agarose at room temperature for 10 min. The supernatant (Flow through) was stored for later analysis. The agarose resin was washed three times in 10 volumes PBS. The first wash fraction (Wash) was stored for later analysis. Biotinylated proteins bound to the streptavidin resin were eluted by boiling for 10 min at 95°C in 20 μl SDS loading buffer (Eluate).

### SDS PAGE, Dot Blot, Western Blot and silver staining

*R*. *typhi* lysate (containing 1–5 μg protein) was mixed with 1/5 volume of 5×SDS loading buffer (45 mM Tris-base, 1.8% SDS, 4.5% glycerin, 0.1 M dithiothreitol, 0.009% bromophenol blue), and the mixture was incubated for 5 min at 95°C. The preparations were either dripped onto a nitrocellulose membrane (GE Healthcare, Freiburg, Germany) (Dot Blot) or separated by electrophoresis through 10% SDS polyacrylamide separating gels with 4.8% polyacrylamide stacking gels at 80 V for 10 min and then at 120 V for about 1 h in a gel chamber (Peqlab Biotechnologie GmbH, Erlangen, Germany). The separated proteins in the gels were electroblotted onto nitrocellulose membranes with 1 mA per cm^2^ of membrane for 45 min or 75 min, respectively. The Blots were blocked for 1–2 h at room temperature or overnight at 4°C with 4% dry milk in Tris-buffered saline (TBS; 20 mM Tris-HCl (pH 7.5), 136 mM NaCl, 1% Tween 20) and washed with TBS. Sera or antibodies were applied to the blots which were then incubated overnight at 4°C. Sera and antibodies were diluted in TBS. Sera were diluted 1:200. The BNI52 antibody was used at a concentration of 1 μg/mL and the penta-His antibody (Qiagen, Hilden, Germany) was diluted 1:1000. After three washes in TBS (10–15 min each), the blots were incubated for 1h with peroxidase-conjugated goat anti-human IgG/A/M (1:1000 in TBS; ThermoFisher Scientific, Darmstadt, Germany) or goat anti-mouse IgG (1:1000 in TBS, Dako Deutschland GmbH, Hamburg, Germany). The blots were again washed three times (10–15 min each) in TBS and enhanced chemiluminescence Western Blotting Substrate (ThermoFisher Scientific, Darmstadt, Germany) was used for detection of bound conjugate. The PageRuler Plus prestained protein ladder (ThermoFisher Scientific, Darmstadt, Germany) was used to estimate the molecular masses of the separated antigens. For the staining of proteins in gels the FireSilver staining kit (Proteome Factory AG, Berlin, Germany) was used according to the manufacturer’s protocol. 2D gel electrophoresis, silver staining of 2D gel and blotting of the separated antigens on a membrane were performed by the Proteome Factory AG (Berlin, Germany), using a bacterial lysate with a protein concentration of 150 μg.

### Enzyme-linked immunosorbent assay (ELISA)

*R*. *typhi* lysates that were either obtained from *R*. *typhi* prior to the infection of BALB/c CB17 SCID mice or re-isolated *R*. *typhi* from these animals (*R*. *typhi*^SCID^). Lysates were diluted in coating buffer (10 mM NaHCO3, pH 9.6), and 50 μl of these lysates (approximately 100 μg protein/mL) was used for coating of ELISA microplates (Greiner Bio-One, Frickenhausen, Germany) overnight at 4°C. After blocking with 50 μl 1% BSA in PBS for 2h, 50 μl of the BNI52 antibody (1 μg/mL in PBS) was added for 2h at room temperature. Plates were washed two times with 200 μl PBS, followed by incubation with 50 μl goat anti-mouse-HRP conjugate (1:200 in PBS; Dako Deutschland GmbH, Hamburg, Germany) for additional 2h. Plates were finally washed three times with 200 μl PBS. Bound antibody was visualized by incubation with 50 μl substrate solution (100 mM NaH_2_PO_4_, 0.4 mM TMB, 0.003% H_2_O_2_). The reaction was stopped by addition of 25 μl 2 M H_2_SO_4_ and quantified photometrically at 450 nm.

### Immunoprecipitation

Protein G sepharose (2 mL; GE Healthcare, Freiburg, Germany) was equilibrated with binding buffer (10 mM sodium phosphate, 50 mM ammonium bicarbonate, pH 7). After that, 130 μg BNI52 antibody and *R*. *typhi* lysate with a protein concentration of 1.5 mg/mL were added to the column and incubated overnight at 4°C. The column was washed five times in 4 mL binding buffer. The proteins were eluted five times with 500 μL 0.1 M glycine buffer, pH 2.7 and immediately neutralized with 75 μl 1 M Tris-HCl, pH 9. All washing steps were carried out by centrifugation of the column material at 430 ×g.

### Mass spectrometry

For qualitative and quantitative proteome analysis, defined protein spots from 2D gel electrophoresis (2DE) or whole SDS-PAGE pathways were subjected to tryptic in gel digestion as described by Kwiatkowski et al. [40]. Briefly, proteins were first reduced with dithiothreitol (c = 10 mM, dissolved in 100 mM NH_4_HCO_3_), followed by alkylation with iodacetamide (c = 55 mM, dissolved in 100 mM NH_4_HCO_3_) and overnight digestion with trypsin (c = 13ng/μL, dissolved in 50 mM NH_4_HCO_3_).

For quantitative analysis of protein contents in whole cell lysates of *R*. *typhi* prior to the infection of mice and bacterial reisolates from SCID mice (*R*. *typhi*^SCID^), proteins were reduced using dithiothreitol (c = 20 mM, dissolved in 1% SDC, 100 mM ammonium hydrogen carbonate buffer) at 56°C for 30 min, followed by alkylation with iodoacetamide (c = 60 mM, dissolved in 1% SDC, 100 mM ammonium hydrogen carbonate buffer) at room temperature in the dark for 30 min and overnight digestion with trypsin (1:100 enzyme to protein ratio). SDC was precipitated by acidifying with formic acid to a final concentration of 2%. The samples were centrifuged at 20000xg for 5 min and the supernatant transferred to a new tube. Peptides were desalted using Oasis HLB cartridges (Waters, Cat.No: WAT094225) as per manufacturers instruction. Desalted peptides were dried in vacuo and stored at -20°C until measurement.

The dried peptide samples were dissolved in 20 μL 0.1% formic acid (FA) and analyzed by LC-MS using a orbitrap tribrid mass spectrometer (Orbitrap Fusion, San Jose, CA, USA) as described in detail earlier [40]. For protein identification from the 2DE spots, peptides were separated by reversed phase chromatography (Acclaim PepMap 100, C18, 75 μm × 250 mm, 2 μm, 100 Ǻ, Thermo Scientific, Bremen, Germany, buffer A: 0.1% FA in HPLC-H_2_O; buffer B: 0.1% FA in acetonitrile) using gradient from 2–30% buffer B in 30 min. For quantitative proteome analysis from the SDS-PAGE, peptides were separated by reversed phase chromatography using a gradient from 2–30% buffer B in 90 min. Mass spectrometric analysis were performed in data dependant acquisition mode (DDA) using top speed mode, a HCD collision energy of 28%, an intensity threshold of 2e5 and an isolation width of 1.6 m/z. Every second a MS scan was performed over a m/z range from 400–1500, with a resolution of 120000 FWHM at m/z 200 (transient length = 256 ms, maximum injection time = 50 ms, AGC target = 2e5). MS/MS spectra were recorded in the ion trap (scan-rate = 66 kDa/s, maximum injection time = 200 ms, AGC target = 1e4).

LC-MS raw data from the 2DE spots were processed with MaxQuant (version 1.5.2.8). For identification MS/MS spectra were searched with Andromeda search engine against the *Rickettsia typhi* swiss-prot database (*Rickettsia typhi* (strain ATCC VR-144 / Wilmington), *Rickettsia conorii* (strain ATCC VR-613 / Malish 7) and *Rickettsia prowazekii* (strain Madrid E), www.uniprot.org) and a contaminant database (298 entries). The searches were performed using the following parameters: precursor mass tolerance was set to 10 ppm and fragment mass tolerance was set to 0.5 Da. Furthermore, two missed cleavages were allowed and a carbamidomethylation on cysteine residues as a fixed modification as well as an oxidation of methionine residues as a variable modification. Peptides and proteins were identified with a FDR of 1%. Proteins were kept as correctly identified if at least one unique peptides was identified.

For quantitative proteome analysis, LC-MS raw data from the SDS-PAGEs and whole cell lysates were processed with MaxQuant (version 1.5.2.8) as described above. Proteins were quantified with the MaxLFQ algorithm considering only unique peptides and a minimum ratio count of two. Bioinformatics data processing (log2-transformation, normalization), statistical analysis (two-sided Student’s T-test using a permutation-based FDR with an adj. p-value cutoff of 0.05) and data visualization, was performed with Perseus.

### Cloning and expression of GroEL

The *R*. *typhi GroEL* gene (RT0617) was amplified from genomic *R*. *typhi* DNA via PCR using the primers GroEL-Ndel-F 5´-ATCATATGACAACGAAACTTATTAAACACGGGTCA-3´ and GroEL-BamHI-R 5´-TAGGATCCTTAGAAGTCCATACCACCCATAC-3´, introducing a 5´ NdeI and 3´ BamHI restriction site. For one 25 μl reaction 100 ng *R*. *typhi* DNA, 25 pmol of each primer, 25 μM MgCl_2_, 2 μM dNTPs, 1x Taq DNA polymerase buffer and 1 unit Taq DNA polymerase (Thermo Fisher Scientific) were used. The PCR program consisted of an initial denaturation step at 94°C for 5 min, 30 cycles of denaturation at 94°C for 45 sec, annealing at 55°C for 45 sec and extension for 1 min at 72°C, followed by a terminal extension step for 10 min at 72°C. Amplified fragments were ligated into the pCR2.1 TA cloning vector (Life technologies, Darmstadt, Germany) according to the manufacturer’s instructions followed by directional subcloning between the NdeI and BamHI restriction sites of the pJC45 expression vector that fuses a N-terminal 10×His tag to the recombinant protein. The generated plasmid pJC45-GroEL (15 ng) was transformed into chemically competent *E*. *coli* BL21(DE3) in KCM solution (100 mM KCl, 30 mM CaCl_2_, 50 mM MgCl_2_) for 20 min on ice and additional 10 min at room temperature before plating on LB agar plates.

Transformed *E*. *coli* BL21(DE3) bacteria were grown in 250 mL LB medium with Ampicillin (100 μg/mL) at 37°C until the value of 0.4 to 0.6 at OD_600_ was reached. Expression of the GroEL protein was induced by adding isopropyl β-D-1-thiogalactopyranoside (IPTG; Sigma-Aldrich, Germany) in a final concentration of 0.5 mM. After an incubation period of 3 h at 37°C, the bacteria were washed with LB medium. The bacterial pellet that was obtained after centrifugation (3220×g, 20 min, 4°C) was resuspended in 10 mL PBS/10 mM imidazole followed by sonification in a Branson Sonifier (twenty times for 30 sec/ output control 1.5/ duty cycle 40). After centrifugation (3220xg, 20 min, 4°C), the supernatant was used for the purification of GroEL. The GroEL protein was purified under native conditions using 12 mL nickel-charged nitrilotriacetic acid (Ni-NTA) agarose column (QIAGEN, Hilden, Germany). After equilibration of the Ni-NTA agarose with PBS/ 10 mM imidazole, the lysate was incubated with the Ni-NTA agarose overnight at 4°C with agitation. The column was washed five times with 2 volumes of PBS/25mM imidazole and the proteins were eluted with 2 mL PBS/250 mM imidazole. The buffer was then replaced by PBS using PD-10 columns (GE Healthcare, Freiburg, Germany) according to the manufacturer’s instructions.

### Cloning and expression of overlapping OmpB fragments

The DNA encoding for the p32 peptide of OmpB (OmpB_1329-1645_) and the surface-exposed portion of the OmpB protein (RT0699; OmpB_1-1328_) was codon-optimized for the expression in *E*. *coli* or eukaryotic cells, respectively. Three overlapping fragments of the mature OmpB protein (OmpB F1: aminoacids 2–460, OmpB F2: amino acids 440–901, OmpB F3: amino acids 881–1330) were designed and a a 6×His tag was fused to the N-terminus of all recombinant proteins. The coding DNAs were synthesized by Invitrogen/ThermoFisher Scientific, Darmstadt, Germany. The p32 peptide encoding DNA was cloned into the pRSET A vector for bacterial expression via BamHI and EcoRI (Thermo Fisher Scientific). The DNAs for the expression of the overlapping fragments of mature OmpB were cloned into the LeGOiC2 expression vector for eukaryotic expression via the same restriction sites [41] (http://lentigo-vectors.de/; kindly provided by Christopher Weber, University Medical Center, Hamburg). The LeGOiC2 vector couples the expression of the recombinant proteins to the expression of the red fluorescent protein mCherry via an IRES sequence. T4 DNA ligase (ThermoFisher Scientific, Darmstadt, Germany) was used for ligations. Vectors were transformed into chemically competent *E*. *coli* Top10 for plasmid conservation or *E*. *coli* BL21 (DE3) for protein expression (Life technologies, Carlsbad, USA) in case of the p32 peptide via the KCM method as described above. The LeGOiC2-OmpB plasmids were isolated using the Plasmid Maxi Kit (Qiagen, Hilden, Germany) according to the manufacturer’s instructions and used for the expression of the recombinant OmpB fragments in HEK293T cells. For the transfection 2.8 μg LeGO-iC2-OmpB-F1 or–OmpB-F2 or–OmpB-F3 or as a control LeGO-iC2 without insert, 1.4 μg pMDLg/pRRE, 0.7 μg pRSV-Rev and 0.28 μg pCMV-VSV-G were incubated for 15 min in 140 μL HEPES buffered saline (Sigma-Aldrich, St. Louis, USA) with 125 mM calcium chloride. 1×10^5^ HEK-293 T cells were plated in 6-well plates in 1.4 mL DMEM high Glucose medium (Gibco/ThermoFisher Scientific, Darmstadt, Germany) supplemented with 15% FCS, 2 mM L-glutamine, 10 mM HEPES, and 25 μM chloroquine (Sigma-Aldrich, Germany) and incubated with one reaction mix that was added drop-wise to the cell culture. After six hours at 37°C and 5% CO_2_ the medium was replaced by medium without chloroquine and the cells were further incubated. Transfected cells were detected microscopically by the expression of mCherry. Cells with a transfection rate of 70–100 percent were used for lysate production. Cells were resuspended in lysis buffer 1, incubated for 60 min on ice, and the cells were disrupted in a Branson Sonifier (five times for 30 seconds/ Output control 2/ Duty cycle 40). Cell debris was removed through centrifugation (3220×g, 10 min, 4°C) and the supernatant was used as lysate.

### Infection and immunization of mice and collection of sera

C57BL/6, BALB/c and CB17 SCID mice (CB17/lcr-PrkdcSCID/lcrlcoCrl) were infected with *R*. *typhi* as described before [42]. Blood was taken by cardiac puncture after euthanasia with CO_2_ seven to 14 days post infection. Serum samples were obtained by coagulation for 15–20 min at room temperature followed by centrifugation for 10 min at 5654×g. For the immunization of a BALB/c mouse the animal was treated two times with 1×10^8^ copies of heat-inactivated *R*. *typhi* (30 min, 56°C) in a three weeks intervall. Serum was taken 7 days after the second immunization.

### Histological staining of *R*. *typhi* in lung sections

Lungs from infected BALB/c CB17 SCID mice were prepared on d3 and d14 post infection, fixed in 4% formalin in PBS and embedded in paraffin. Sections were deparaffinized by first heating at 63°C for 30 min in a heating cabinet followed by treatment with Xylol for 30 minutes and serial treatment with EtOH (3x 100% EtOH, 3x 96% EtOH, 80% EtOH, 70% EtOH), each step performed for 3-5 min. Slides were finally washed in H_2_O and boiled for 30 min in 10 mM citrate buffer (10 mM sodium citrate, 0.05% Tween20, pH6) for antigen retrieval. A Ventana Benchmark XT apparatus (Ventana Medical Systems, Tucson, USA) was used for the stainings. Antibodies were diluted in 5% goat serum (Dianova, Hamburg, Germany) in Tris-buffered saline (TBS, pH7.6) and 0.1% Triton-X 100 in antibody diluent solution (Zytomed, Bargteheide, Germany). For the detection of *R*. *typhi* either BNI52 (1 μg/mL) or serum from a patient (1:100) were used and slides were incubated for 1h with the primary antibodies. Anti-human or anti-mouse Histofine Simple Stain Mouse Max peroxidase-coupled antibodies (Nichirei Biosciences, Japan) and Ultraview Universial DAP detection kit (Ventana Medical Systems, Tucson, USA) were used for detection. Slides were analyzed on a BZ9000 microscope (Keyence, Neu-Isenburg, Germany).

### Isolation of *R*. *typhi* from SCID mice (*R*. *typhi*^SCID^)

1.4×10^6^ L929 cells were seeded in 25 cm^2^ culture flasks (Greiner Bio-One, Frickenhausen, Germany) and γ-irradiated (1966 rad at 560 sec) one day before use. Seven to 14 days post infection spleens from CB17 SCID mice were isolated and passed through a cell strainer (70 μm) in PBS. One third of each of the spleen cell suspension was added to L929 cells in RPMI 1640 medium supplemented with 10% FCS, 2 mM L-glutamine, and 10 mM HEPES without antibiotics. The medium of the L929 cells was changed one day later. After 3 to 6 days one half of the L929 cells were added to γ-irradiated L929 cells and incubated for 5 to 7 days. Further infections were carried out by adding 5 or 50 μL of infected L929 cells to γ-irradiated L929 cells and incubation for 5 to 7 days. Bacterial stocks were prepared as described previously [12] after 5 passages and used for analyses of protein expression.

### Generation of bone marrow-derived macrophages (bmMΦ) and dendritic cells (bmDC)

Bone marrow was prepared by rinsing the femur and tibia of the hind legs of BALB/c mice with PBS. For the generation of bmDC erythrocytes were lyzed by incubating the bone marrow for 5 min in erythrocyte lysis buffer (144 mM NH_4_Cl, 10 mM Tris/HCl, pH7.5) at room temperature. Cells were centrifuged (1200 rpm, 5 min) after adding 20 mL RPMI1640/10% FCS and washed two times in cell culture medium. Purified bone marrow cells (2×10^6^) were plated into petri dishes (Sarstedt, Nümbrecht, Germany) and cultured in RPMI1640/10% FCS supplemented with 20 ng/mL GM-CSF for 9–10 days to obtain bmDC. Thereby, medium was exchanged every three days. For the generation of bmMΦ erythrocyte lysis was not performed. Bone marrow cells (2×10^6^) were plated into bacterial culture dishes (Sarstedt, Nümbrecht, Germany) in IMDM medium (PAA laboratories, Cölbe, Germany) additionally supplemented with 30% L929 fibroblast cell culture supernantant and 5% horse serum. Medium was exchanged every three days and cells were used after 10–12 days of culture.

### Infection of bmMΦ, bmDCs, Z232 macrophages and L929 cells with *R*. *typhi* and immunofluorescence staining

Cells were seeded into Permanox 8well chamber slides (Lab Tek/ThermoFisher Scientific, Darmstadt, Germany) to build a cell monolayer (4×10^5^ in case of bmMΦ and bmDC; 3×10^5^ in case of Z232 macrophages and L929 fibroblasts). bmMΦ and bmDC were infected with 4×10^6^ *R*. *typhi* (10 particles per cell) in the presence of either 8 μg/mL BNI52 or IgG3 isotype antibody (clone B10; SouthernBiotech, Birmingham, USA) in a total volume of 240 μl PRMI1640 cell culture medium. Cells were incubated for 24h, carefully washed once in 500 μl PBS and then fixed in ice-cold acetone:ethanol (2:3) at -20°C for 10 min. For the detection of the bacteria the Fc receptors were blocked with mouse serum (1:100 in PBS, 20 min, room temperature) and then stained with patient serum (1:100; 30 min, room temperature) and goat anti-human IgG-FITC secondary antibody (1:200, 30 min, RT; H10101C, ThermoFisher Scientific, Darmstadt, Germany). In addition, nuclei were stained with 4,6-diamidin-2-phenylindol (DAPI) (1:1000 in PBS, 30 min room temperature; Sigma-Aldrich, Germany). Z232 and L929 cells were infected with 5 *R*. *typhi* particles per cell in the presence of indicated amounts of BNI52 or isotype antibody. In one experiment Z232 were pretreated with increasing amounts of CohnII human IgG immunoglobulin fraction (Sigma-Aldrich, Munich, Germany) to block Fc receptors prior to the addition of 10 μg/mL BNI52 or IgG3 isotype antibody. Cells were then incubated for 3h at 37°C, carefully washed once in 500 μl PBS and then fixed in ice-cold acetone:ethanol (2:3) at -20°C for 10 min. Staining of the bacteria was performed with BNI52 (1:100 in PBS; 30 min, room temperature), goat anti-mouse IgG3-FITC (1:200 in PBS, 30 min, room temperature; SouthernBiotech, Birmingham, USA) and anti-FITC-Alexa488 (1:1000 in PBS, 30 min, room temperature; Sigma-Aldrich, Germany). Nuclei were stained with DAPI (1:1000 in PBS, 30 min, room temperature; Sigma-Aldrich, Munich, Germany). Slides were covered with Permafluor (ThermoFisher Scientific, Darmstadt, Germany) and analyzed by immunofluorescence microscopy on a BZ9000 microscope (Keyence, Neu-Isenburg, Germany).

### Quantitative PCR (qPCR)

Detection of *R*.*typhi* DNA was performed by amplification of a 137-bp fragment of the *prsA* gene (RT_RS02795) as described before [12]. The forward primer 5´-ACA GCT TCA AAT GGT GGG GT-3´ and reverse primer 5´-TGC CAG CCG AAA TCT GTT TTG-3´ were used in a standard SYBR green qPCR. Titrated amounts of the plasmid vector pCR2.1-PrsA served as a standard for the calculation of bacterial copies. The 10 μl reaction mix contained 1.5 mM MgCl_2_, 0.175 mM dNTPs, 100 nM of each primer, 0.05x SYBR green I nucleic acid gel stain (Sigma Life Sciences, Deisenhofen, Germany) and 0.25 U HotStar Taq DNA Polymerase (Qiagen, Hilden, Germany). A Rotor Gene 6000 (QIAGEN, Hilden, Germany) was used for the PCR reaction at following conditions: 15 min of preheating at 95°C, 40 cycles of denaturing (94°C, 20 sec), primer annealing (53°C, 30 sec) and elongation (72°C, 20 sec).

### Statistical analysis

Mann-Whitney U test was used for statistical analysis employing GraphPad Prism software (GraphPad Software Inc., San Diego, USA).

## Results

### The BNI52 antibody specifically binds to TG rickettsiae

**Fig 1:**
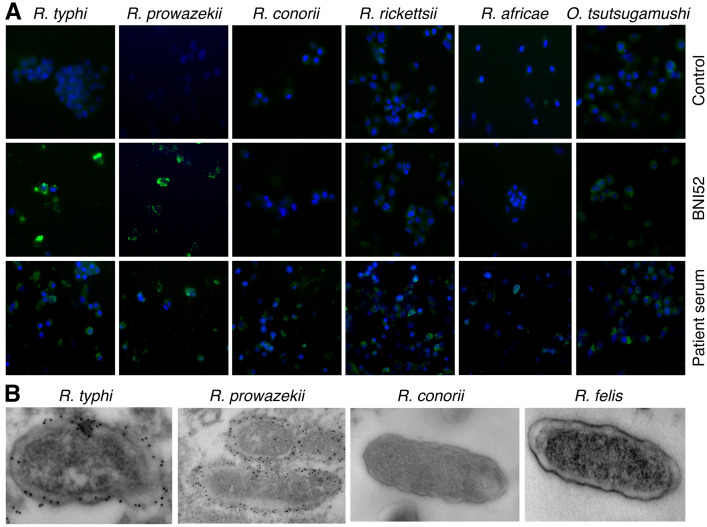
BNI52 specifically detects TG rickettsiae. (A) L929 cells were infected with the indicated TG and SFG rickettsiae or *Orientia tsutsugamushi* and stained with isotype antibody as a negative control (upper panel), BNI52 (middle panel) or serum from patients suffering from the respective infection (lower panel). Bacteria are shown in green. Nuclei were stained with DAPI (blue). (B) L929 cells were infected with TG rickettsiae (*R*. *typhi*, *R*. *prowazekii*), SFG rickettsiae (*R*. *conorii*) or transitional rickettsiae (*R*. *felis*), stained with BNI52 and gold-labeled secondary antibody and analyzed by electron microscopy. The antibody binds to the TG rickettsiae *R*. *typhi* and *R*. *prowazekii* but not to the SFG rickettsiae *R*. *conorii*, *R*. *rickettsii* and *R*. *africae* or to *R*. *felis*.

This study was initially intended to characterize the BNI52 antibody. To determine the specificity of the BNI52 antibody, immunofluorescence stainings and electron microscopic studies of L929 cells and XTC-2 cells that were infected with different SFG rickettsiae (*R*. *conorii*, *R*. *rickettsii*, and *R*. *africae*), the TG rickettsiae *R*. *typhi* and *R*. *prowazekii*, the transitional group rickettsial species *R*. *felis* and *O*. *tsutsugamushi* were performed. After fixation cells were stained with the BNI52 antibody and a fluorescence-labeled or gold-labeled secondary antibody for electron microscopy, respectively. Immunofluorescence stainings (Fig 1A) as well as electron microscopy studies (Fig 1B) showed that the antibody BNI52 specifically binds to both TG rickettsiae members but does not recognize rickettsiae of the SFG group, *R*. *felis* or *O*. *tsutsugamushi*.

### The BNI52 antibody recognizes immunodominant *R*. *typhi* antigens of ±30 kDa

**Fig 2:**
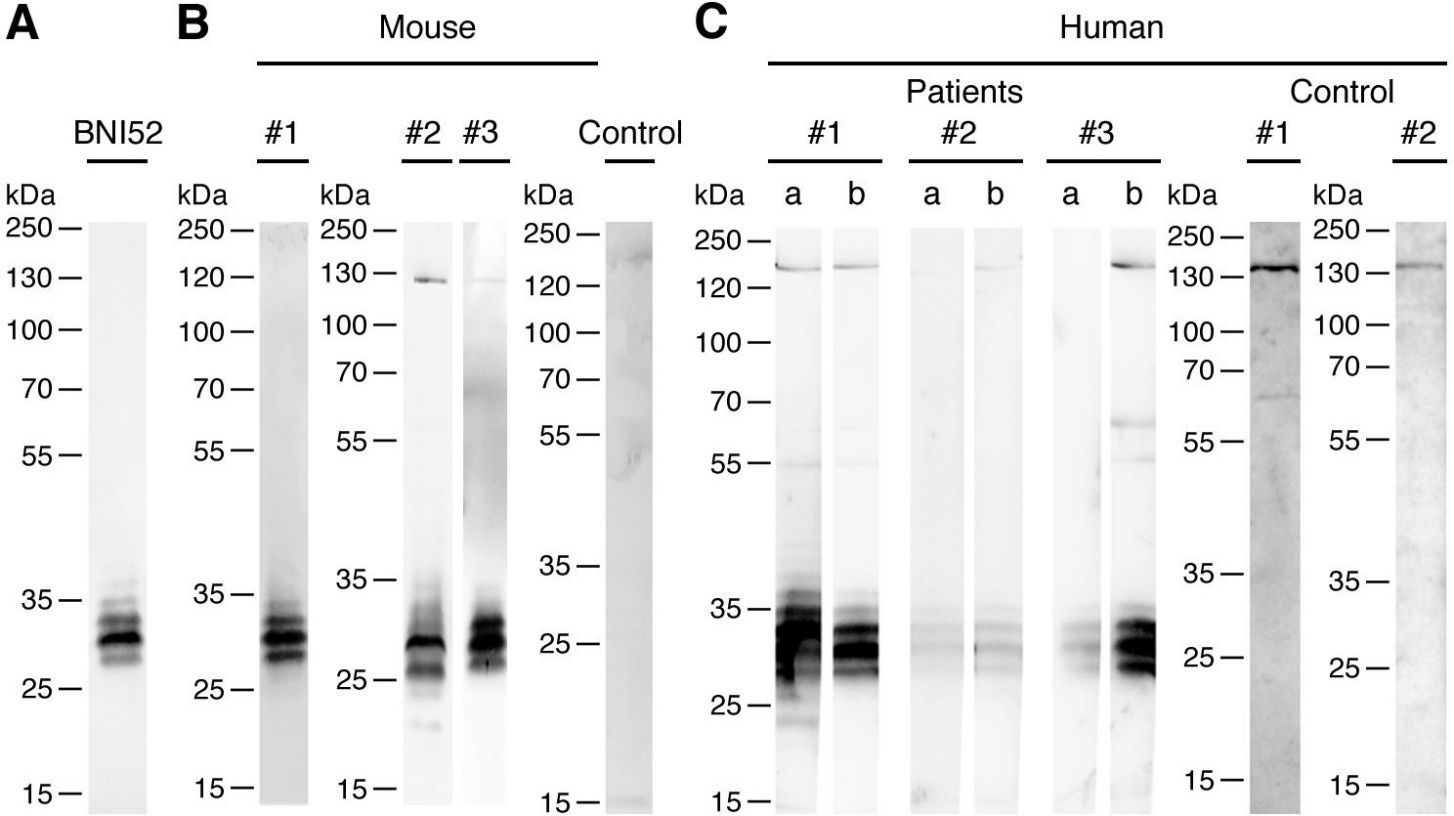
BNI52 detects immunodominant peptides of 25–35 kDa. **(A-C)** Proteins in the lysate of *R*. *typhi* were seperated by SDS PAGE and blotted on a nitrocellulose membrane. **(A)** The membrane was incubated with the monoclonal BNI52 antibody and HRP-labeled anti-mouse Ig secondary antibody. **(B)** Western Blots from *R*. *typhi* lysate were detected with serum from a BALB/c mouse immunized with inactivated *R*. *typhi* (#1), serum from mice that were infected with viable *R*. *typhi* (#2: BALB/c; #3: C57BL/6) or serum from a naive BALB/c mouse (Control) and HRP-labeled anti-mouse Ig secondary antibody. **(C)** Western Blots from *R*. *typhi* lysate were detected with serum from patients who acquired the infection with TG rickettsiae, most likely *R*. *typhi*, in different countries (#1: Greece, #2: Laos/Cambodia; #3: Thailand) and HRP-labeled anti-human Ig antibody. Patient serum was taken at different points in time (a: early, b: late). Serum from two healthy indivuals was used as a control (Control #1 and #2). BNI52 as well as antibodies in the sera from patients and immunized or infected mice predominantly recognize three to five antigen bands ranging from 25 to 35 kDa.

In the next step, Western Blots with the lysate of *R*. *typhi* were performed and membranes were incubated with the BNI52 antibody and HRP-labeled secondary antibody. Three to four antigen bands with the size of 25 to 35 kDa were detected in the lysate of *R*. *typhi* with the BNI52 antibody, whereby a 30 kDa antigen band was dominantly recognized (Fig 2A). Further Western Blots were carried out to investigate whether the antigen is also recognized by antibodies in sera from BALB/c mice that were immunized with inactivated *R*. *typhi* and from BALB/c and C57BL/6 mice that were infected with viable *R*. *typhi* (Fig 2B) as well as from human patients infected with TG rickettsiae (Fig 2C). Therefore lysates of *R*. *typhi* were again separated by SDS PAGE and applied to Western Blots that were incubated with the indicated sera followed by a HRP-labeled secondary antibody. Sera from non-infected individuals were used as a control. Antibodies in the serum from the immunized BALB/c mouse detected antigens of ±30 kDa (Fig 2B, #1) as observed for the detection with the BNI52 antibody. Similar protein bands were observed with serum from infected BALB/c (Fig 2B, #2) and C57BL/6 mice (Fig 2B, #3) both of which additionally detected a protein of approximately 130 kDa. No antigens were detected by antibodies in the serum of a naive BALB/c mouse (Fig 2B, Control).

In case of human sera, sera from different points in time after infection (a: earlier, b: later) were used. In addition, the patients acquired the infection in different countries (#1: Greece, #2: Laos/Cambodia; #3: Thailand). All patients had high IgG/M/A titers (1:640–1:10240) with patient #2 showing the lowest titer. Interestingly, antibodies in the sera from all patients detected three to four antigen bands with the size of 25–35 kDa that resemble the bands detected by the BNI52 antibody as well as by antibodies in the sera from immunized or infected mice. It is therefore likely that these antigens are identical to those recognized by BNI52. Antibodies in the patient sera additionally detected rather weak bands of approximately 130 kDa, 60 kDa and 55 kDa (Fig 2C) of which only the 130 kDa band was also recognized by antibodies in the serum from an infected mouse (Fig 2B, #2) but not the immunized mouse (Fig 2B, #1). However, a protein band of similar size was also detectable with sera from non-infected individuals (Fig 2C, Control #1 and #2). Together, these observations show that the ±30 kDa antigens that are recognized by BNI52 and patient antibodies are immunodominant during the infection with *R*. *typhi*, and that the antibodies recognize linear epitopes as the Western Blots were performed under denaturing conditions.

**Fig 3:**
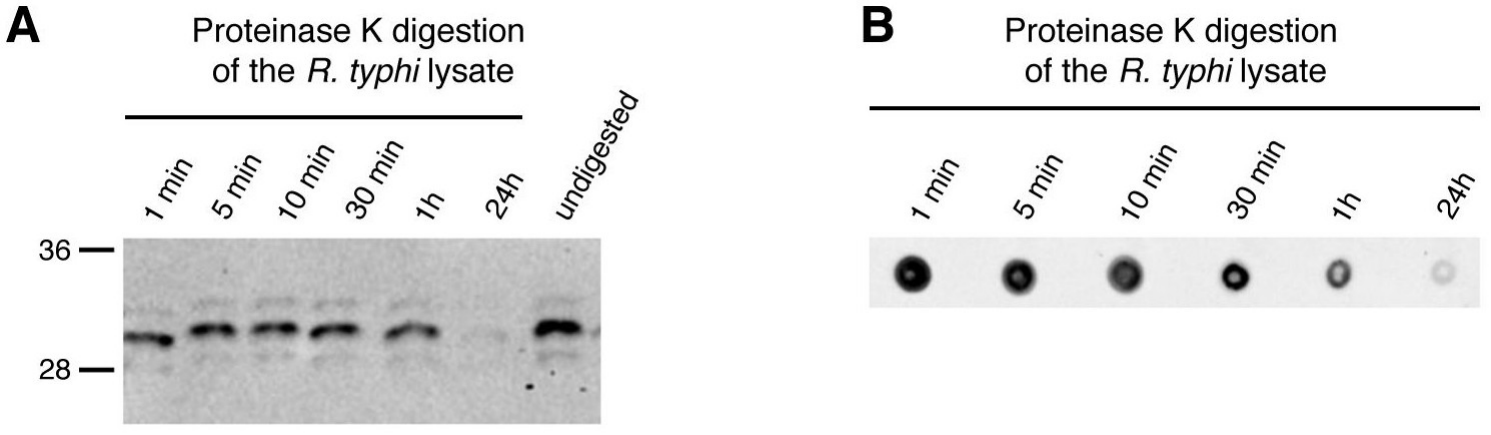
The antigen recognized by BNI52 is a protein. (**A, B**) Proteins in the lysate of *R*. *typhi* were digested with Proteinase K for indicated periods. Control bacterial lysate was left undigested. **(A)** A Western Blot and **(B)** a Dot blot was performed. The membranes were incubated with the BNI52 antibody followed by the incubation with a HRP-labeled secondary antibody. The antigen was no longer detectable after 24h proteinase K digestion.

To clarify whether the BNI52 antibody detects possible modifications rather than a peptide sequence, lysates from *R*. *typhi* were treated with proteinase K and examined for the binding of the antibody by subsequent Western blotting and Dot blotting. Binding of the antibody declined within 1h and was no longer detectable after 24h of digestion of the lysate in both, the Western blot (Fig 3A) and the Dot Blot (Fig 3B). These results suggest that the antibody binds to a sequence of the peptide backbone of the antigen rather than to other structures that may be attached to the protein.

### The ±30 kDa antigen is surface-exposed

**Fig 4:**
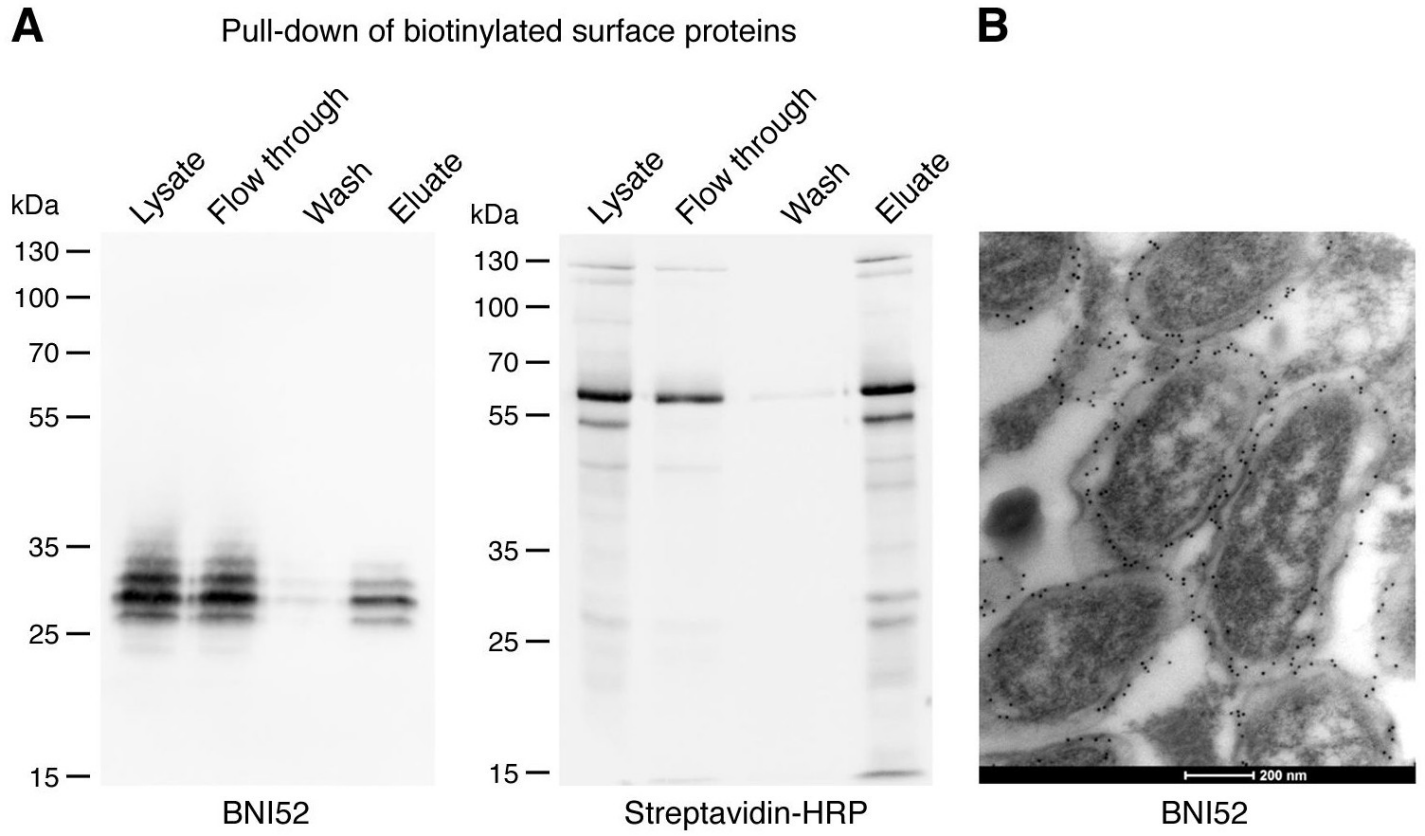
The antigen recognized by BNI52 is surface-exposed. **(A)** *R*. *typhi* surface proteins were biotinylated prior to generating lysate. Biotinylated surface antigens from *R*. *typhi* were then enriched by streptavidine-coupled agarose. Lysates, flow through, wash fraction and eluate were applied to SDS PAGE and Western Blots. Western blots were first incubated with BNI52 (left) and after stripping with streptavidin-HRP (right). The antibody recognizes mainly the protein of 30 kDa and a slightly larger and a slightly smaller protein of about 27 kDa and 32 kDa. **(B)** BNI52 and gold-labeled secondary antibody were used for the staining of *R*. *typhi*-infected L929 cells and electron microscopy. The antigen is located on the outer cell membrane of *R*. *typhi* and in the periplasmic space between outer and the inner membrane.

Analysis of the binding of BNI52 to TG rickettsiae in infected cells by electron microscopy already indicated that the antigen recognized by this antibody seems to be surface-exposed (Fig 1B). In order to determine the location of the antigen that is recognized by the BNI52 antibody, the surface proteins on intact bacteria were biotinylated prior to generating a bacterial cell lysate. Afterwards, the the biotinylated surface proteins were precipitated with streptavidine-coupled agarose. Total cell lysates, flow through, wash fraction and eluates were then applied to SDS PAGE and Western blotting for the detection first with the BNI52 antibody and, after stripping, with streptavidin-HRP. Eleven enriched biotinylated surface proteins (130, 125, 60, 55, 45, 40, 35, 30, 27, 22 and 15 kDa) were detectable with streptavidin-HRP (Fig 4A, right). Within the enriched fraction of biotinylated surface proteins, BNI52 recognized a similar spectrum of proteins as in total lysates (25, 27, 30, 32 kDa) with the 30 kDa protein being the dominant antigen (Fig 4A, left). However, the antigen was also detectable in the flow through in a much higher amount than in the eluate, indicating either insufficient or incomplete biotinylation of the surface proteins or location of major amounts of the antigen within the cell where it is not accessible for biotinylation.

To investigate this in more detail, electron microscopic studies were performed by staining *R*. *typhi* with the BNI52 antibody in infected L929 cells. These studies revealed that the antigenic peptides that are recognized by BNI52 are predominantly exposed on the outer cell membrane of *R*. *typhi* but also located in the intermembrane space between the outer and the inner membrane. The antigen was not detectable in the cytoplasm of the bacteria (Fig 4B).

### BNI52 recognizes the GroEL protein of *R*. *typhi*

**Fig 5:**
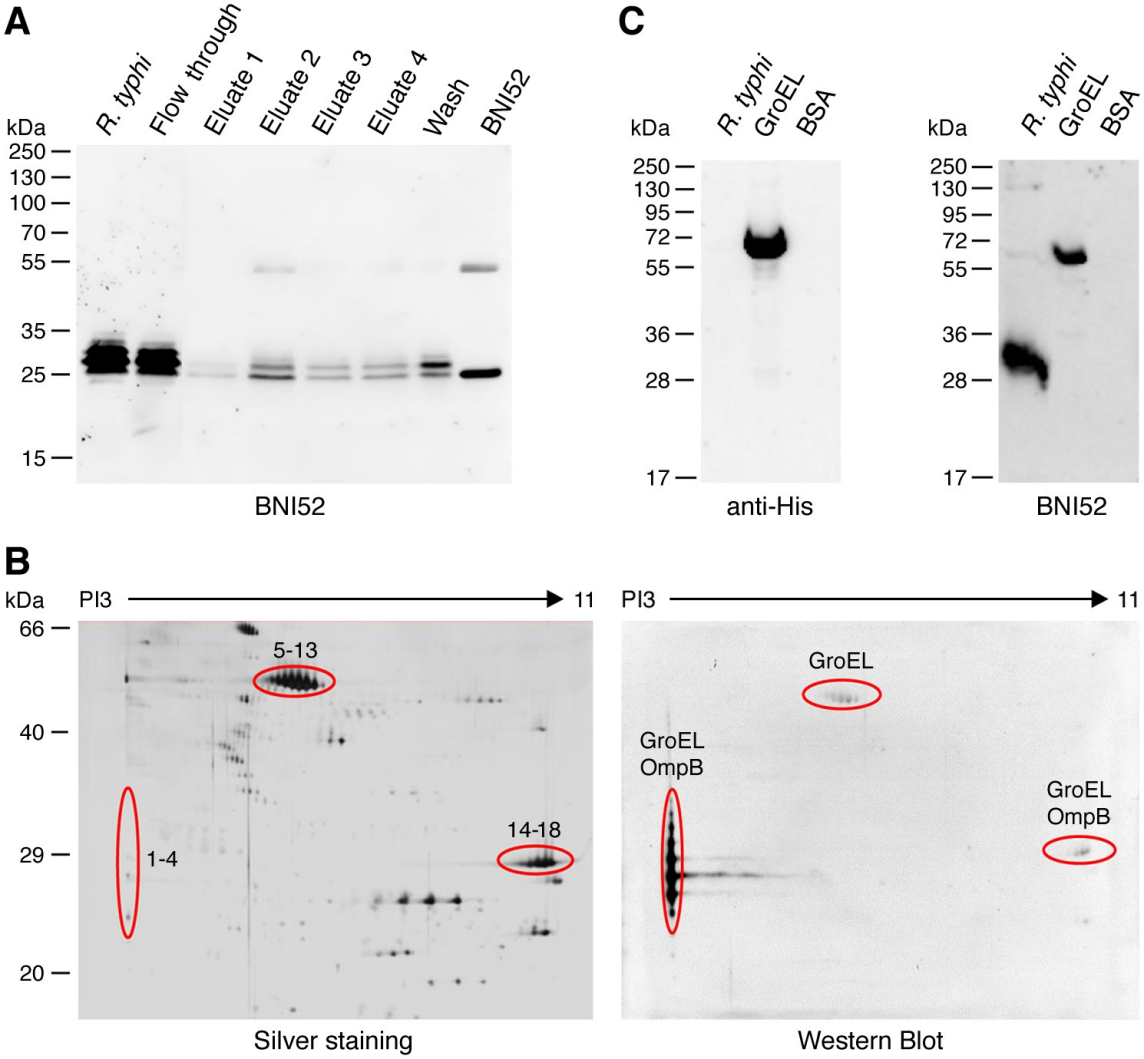
BNI52 binds the GroEL protein of *R*. *typhi*. **(A)** Proteins in the lysate of *R*. *typhi* were precipitated with the BNI52 antibody by a protein A/G column. A Western Blot was performed from total lysate (*R*. *typhi*), flow through, eluates and wash fraction. BNI52 antibody was loaded as a control. The membrane was incubated with the BNI52 antibody. The antibody predominantly precipitates a 30 kDa protein. **(B)** For the identification of the antigen lysate of *R*. *typhi* was separated by two-dimensional SDS PAGE. Gels were silver stained (left) and applied to Western blotting. The membrane was incubated with the BNI52 antibody (right). The indicated protein spots (1–4, 5–13 and 14–18) of the silver stained gels that were recognized by the antibody in Western blots were analyzed by mass spectrometry. These analyses revealed the presence of GroEL in all spots recognized by BNI52. In addition, OmpB peptides were found in the lower molecular weight spots 1–4 and 14–18 but not in the 60 kDa spots. **(C)** His-tagged GroEL protein of *R*. *typhi* was expressed in *E*. *coli*, purified and subjected to Western blotting. Total lysate from *R*. *typhi* and BSA protein were used as a control. The membranes were incubated with a polyhistidine antibody (left) or the antibody BNI52 (right). The BNI52 antibody recognizes the 60 kDa recombinant GroEL protein but only weakly binds to the 60 kDa protein in *R*. *typhi* lysate.

For the identification of the antigen that is recognized by BNI52, the lysate of *R*. *typhi* was incubated with the BNI52 antibody for immunoprecipitation by binding to protein G sepharose and subsequent mass spectrometry analysis. First, the lysate, flow through, wash fraction and eluates were applied to SDS PAGE and subsequent Western Blot. The BNI52 antibody itself was loaded as a control, and the Western Blot was developed with the BNI52 antibody. Two to three antigen bands were detected with the antibody in the eluates of the precipitation, including the dominant 30 kDa protein band, as well as a slightly smaller (27 kDa) and a slightly larger one (32 kDa) (Fig 5A). The smaller band, however, had approximately the same size as the light chain of the BNI52 antibody. A SDS PAGE was then carried out again with the eluates and the gel was silver stained. The 30 kDa and 32 kDa antigen bands were cut out and analyzed by mass spectrometry. In both preparations peptides derived from the GroEL protein as well as from the OmpB protein (outer membrane protein B) of *R*. *typhi* were present.

To achieve a better separation of the proteins, 2D gel electrophoresis was performed, using a bacterial lysate with a high protein concentration of 150 μg. One gel was silver stained (Fig 5B, left) and a second one was used for Western Blot analysis with the BNI52 antibody (Fig 5B, right). Four to seven protein spots with a PI of 3 and a mass of 25–35 kDa were dominantly detected by the antibody (Fig 5B; spots 1–4). To a lower extent the antibody also bound to protein spots with the mass of 60 kDa and a pI ranging from 5 to 7 (Fig 5B; spots 5–13), and to protein spots with the mass of 29 kDa and a more basic pI of about 10 to 11 (Fig 5B; spots 14–18). The indicated protein spots that were detected by the BNI52 antibody were then excised from the corresponding silver stained gel and analyzed by mass spectrometry. Again, the dominantly recognized ±30 kDa acidic protein spots 1–4 and the alkaline protein spots 14–18 with the mass of 29 kDa mainly consisted of peptides from the GroEL protein. In addition, few peptides from the OmpB protein were detected. Exclusively peptides of the GroEL protein were identified in the 60 kDa protein spots 5–13. The GroEL-derived peptides that were found in all ±30 kDa protein spots, acidic as well as alkaline ones, are depicted in S1 Fig and span the region from amino acid 14–308 of the *R*. *typhi* GroEL protein (S1A Fig). Interestingly, the OmpB peptides almost exclusively derived from the p32 peptide of the OmpB protein (S2B Fig) that is eliminated from the protein during the maturation and export process.

These results indicated that the BNI52 antibody likely recognizes the GroEL protein. However, BNI52 may also bind to parts of the OmpB protein or both proteins. Sequence alignment of OmpB and GroEL from *R*. *typhi* did not reveal apparent sequence identities that might serve as an antigenic epitope. To finally clarify whether BNI52 binds to the GroEL or the OmpB protein, these proteins from *R*. *typhi* were recombinantly expressed either in *E*. *coli* (GroEL, p32 peptide) or eukaryotic HEK293T cells (mature OmpB). The GroEL protein was fused to a N-terminal 10×His tag and the OmpB p32 peptide to a N-terminal 6×His tag for the detection and purification of the recombinant proteins. Because the mature OmpB protein is still very large (130 kDa after processing), the protein was expressed as three overlapping fragments (OmpB F1, OmpB F2, OmpB F3) that together encode for the mature surface-expressed OmpB protein. Each OmpB fragment was fused to a N-terminal 6×His tag for detection. The purified reombinant GroEL protein and p32 peptide and the expression of the OmpB fragments in HEK293T cells was analyzed by SDS PAGE and Western blotting. The purified GroEL protein (Fig 5C, left), the purified p32 peptide and the three fragments of mature OmpB present in the lysates from HEK293T cells were detectable with the penta-His antibody (S2B Fig) and, thus, successfully expressed. Subsequently, similar Western Blots of *R*. *typhi* lysate, purified GroEL protein and BSA as a control protein, as well as of purified p32 peptide and lysates from HEK293T cells expressing the three fragments of mature OmpB and non-transfected HEK293T cells as a control were performed and incubated with the BNI52 antibody. The BNI52 antibody bound to the 60 kDa GroEL protein neither to BSA nor the p32 peptide or mature OmpB (Fig 5C, right; S2B Fig). Together these results clearly demonstrate that the BNI52 antibody specifically recognizes the GroEL protein from *R*. *typhi* but not parts of the OmpB protein. They further suggest that GroEL interacts with the OmpB protein during the maturation and export process, and that the OmpB peptides found in the MS analyses are coprecipitated together with GroEL with the BNI52 antibody.

### BNI52 opsonizes *R*. *typhi* for the uptake into phagocytic cells

**Fig 6:**
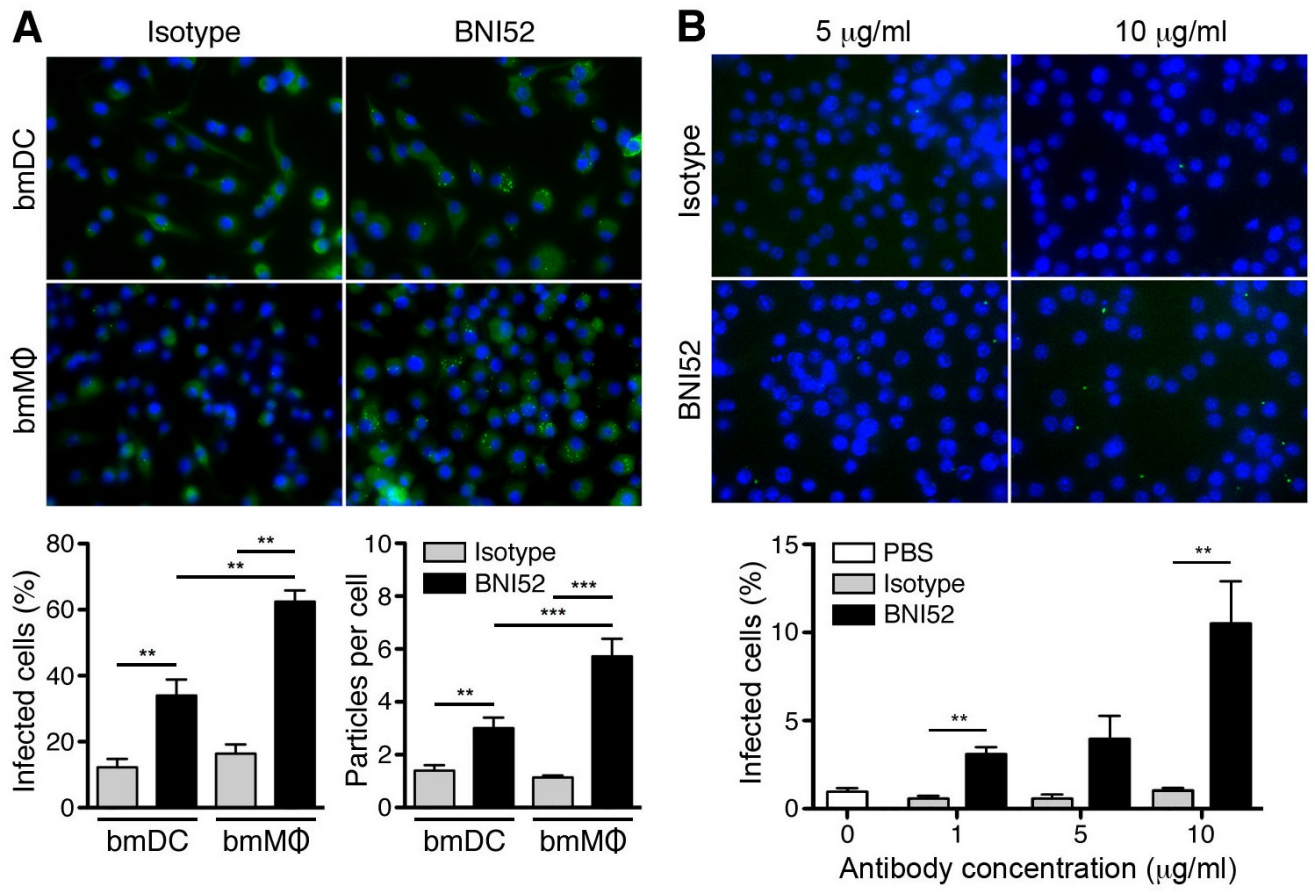
BNI52 enhances the uptake of *R*. *typhi* into phagocytic cells. **(A)** Bone marrow-derived cells dendritic cells (bmDC) and macrophages (bmMΦ) were incubated with *R*. *typhi* (10 particles per cell) in the presence of of either IgG3 isotype antibody or BNI52 (8 μg/mL each) for 24h. Afterwards *R*. *typhi* was stained with patient serum (green) and nuclei were stained with DAPI (blue). An example staining is shown (above). Cells and bacteria were counted from five different views of the slides and the percentage of infected cells (y-axis; below, left) and the amount of *R*. *typhi* particles per cell (y-axis; below, right) were determined and statistically analyzed with Mann Whitney U test (**p<0.01, ***p<0.001). **(B)** Z232 macrophages were incubated with *R*. *typhi* (5 particles per cell) in the presence of increasing amounts of IgG3 isotype antibody or BNI52 for 3h. PBS was used as a control. Cells were then fixed and bacteria were stained with BNI52 (green). Nuclei were stained with DAPI (blue). An example staining is shown (above). From that experiment the percentage of infected cells (y-axis) in five different microscopic views was determined and statistically analyzed with Mann Whitney U test (**p<0.01). The x-axis shows the antibody concentration used (below).

Antibodies can contribute to protection against rickettsial infections by opsonizing the bacteria for the uptake by phagocytic cells. To analyze whether BNI52 has opsonizing properties, dendritic cells (DC) and macrophages (MΦ) were generated from the bone-marrow from BALB/c mice *in vitro* (bmDC, bmMΦ) and incubated with *R*. *typhi* in the presence of either IgG3 isotype antibody or the BNI52 antibody. Cells were analyzed by immunofluorescence staining of the bacteria with patient serum after 24h. Fig 6A shows an example staining of bmDCs and bmMΦ that were incubated in the presence of isotype antibody or BNI52 (Fig 6A, above). The statistical analysis of the percentage of infected cells and the number of engulfed bacteria (Fig 6A, below) shows that the presence of BNI52 leads to a significantly enhanced percentage of both, infected bmMΦ and bmDCs, as well as to enhanced numbers of particles that were taken up per cell compared to control cultures where isotype antibody was added (Fig 6A, below). Thereby, the effect of BNI52 on the uptake of the bacteria by bmMΦ was significantly higher compared to bmDCs.

In addition, the Z232 bmMΦ cell line was employed and incubated the cells with *R*. *typhi* in the presence of increasing amounts of BNI52 or isotype antibody. PBS was used as a control. Fig 6B shows microscopic views of cultures that were incubated with the indicated amounts of either isotype or BNI52 antibody for 3h. The bacterial uptake by Z232 macrophages was comparable in cultures with PBS or isotype antibody while enhanced uptake of *R*. *typhi* was observed in the presence of BNI52 in a dose-dependent and, thus, specific manner (Fig 6B, below). Together, these findings show that BNI52 specifically opsonizes *R*. *typhi* for the uptake by phagocytes The presence of the antibody, however, does not lead to killing of the bacteria in the cells. *R*. *typhi* replicates in MΦ as well as in DCs *in vitro* and the cells finally die.

### GroEL is down-regulated in the absence of adaptive immunity

**Fig 7:**
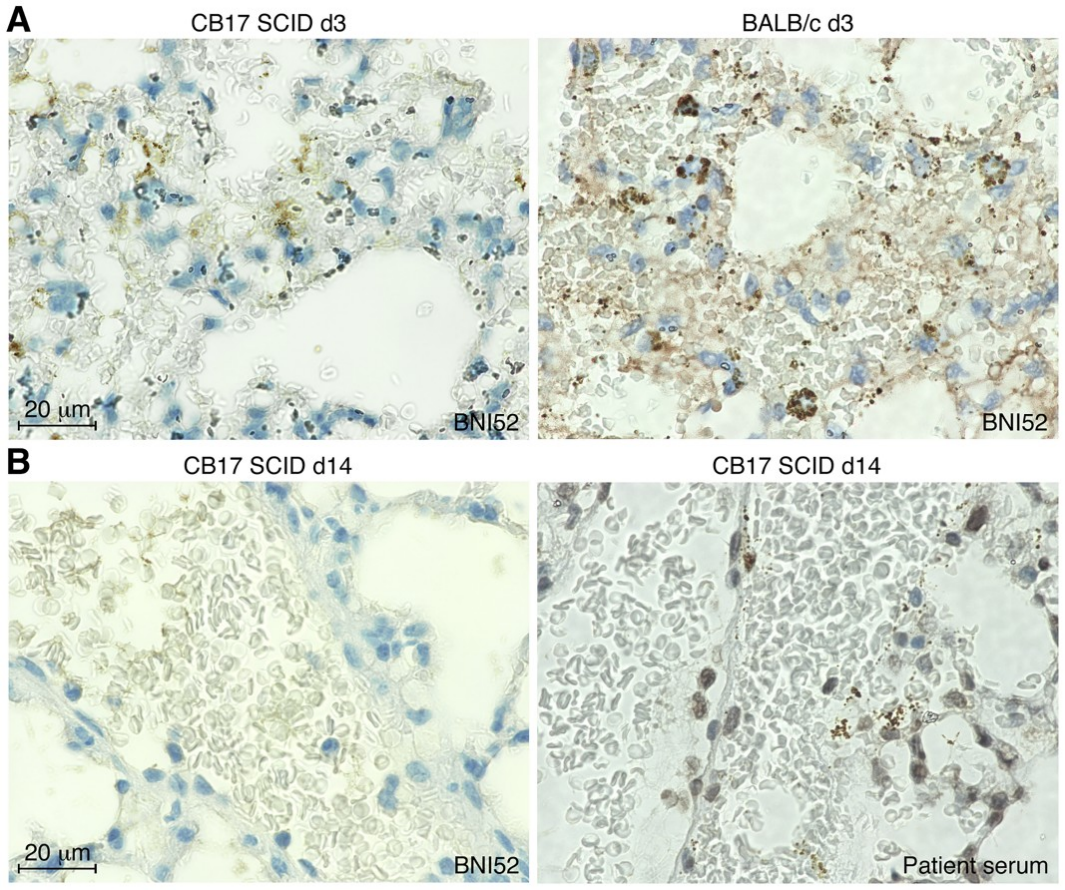
BNI52 does not detect *R*. *typhi* anymore during passage through BALB/c CB17 SCID mice. **(A)** Lung sections were prepared on day 3 post infection from a *R*. *typhi*-infected BALB/c CB17 SCID mouse (left) and a BALB/c wildtype mouse (right) and stained with BNI52. BNI52 detects the bacteria in both sections, although the staining in BALB/c CB17 SCID already appears weaker compared to the staining in the BALB/c wildtype mouse. **(B)** Lung sections from a *R*. *typhi*-infected BALB/c CB17 SCID mouse prepared on day 14 post infection were stained with either BNI52 or patient serum. BNI52 does not detect the bacteria in BALB/c CB17 SCID tissue sections anymore although bacterial loads were high.

In the past years two lethal models of *R*. *typhi* infection have been established employing immunodeficient mice. C57BL/6 RAG1^-/-^ mice develop high bacterial loads in the central nervous system, especially in the brain, and succumb to the infection due to paralysis [12] while the infection of BALB/c CB17 SCID mice with *R*. *typhi* leads to systemic inflammation, high bacterial loads in all organs and death within 20 days [42]. In both models it was striking that it was hardly possible to detect the bacteria in histological stainings of organs from the infected animals with BNI52 although bacterial loads were high as determined by qPCR as described previously [12]. Fig 7A shows an example staining of lung sections of a *R*. *typhi*-infected BALB/c CB17 SCID mouse (left) and an infected BALB/c wildtype mouse (right) on day 3 post infection. At this point in time, *R*. *typhi* was detectable in both sections, although the staining of the bacteria in BALB/c CB17 SCID sections already appeared weaker than in section from BALB/c wildtype mice (Fig 7A). On day 14 post infection, *R*. *typhi* was not detectable anymore in the lungs of BALB/c CB17 SCID mice in stainings with the BNI52 antibody (left) but clearly visible in stainings with serum from a patient (right) (Fig 7B).

Based on these findings we speculated that this could be due to downregulation of the antigen that is specifically recognized by BNI52 while detection of the bacteria with patient serum is still possible because here various antibodies detect a broader range of antigens. To clarify this question *R*. *typhi* was reisolated out of the spleen of infected BALB/c CB17 SCID mice (*R*. *typhi*^SCID^) and cultivated *ex vivo* in L929 cells. *R*. *typhi* was detectable in such infected L929 cells by qPCR analysis and stainings with patient serum but not in immunofluorescence stainings with the BNI52 antibody (S3 Fig). To rule out that this was a random phenomenon, the whole experimental set-up was repeated, this means the isolation of *R*. *typhi* out of the spleen of infected BALB/c CB17 SCID, the cultivation of these isolated bacteria *ex vivo* in L929 cells followed by qPCR and immunofluorescence analysis, with the same results.

**Fig 8:**
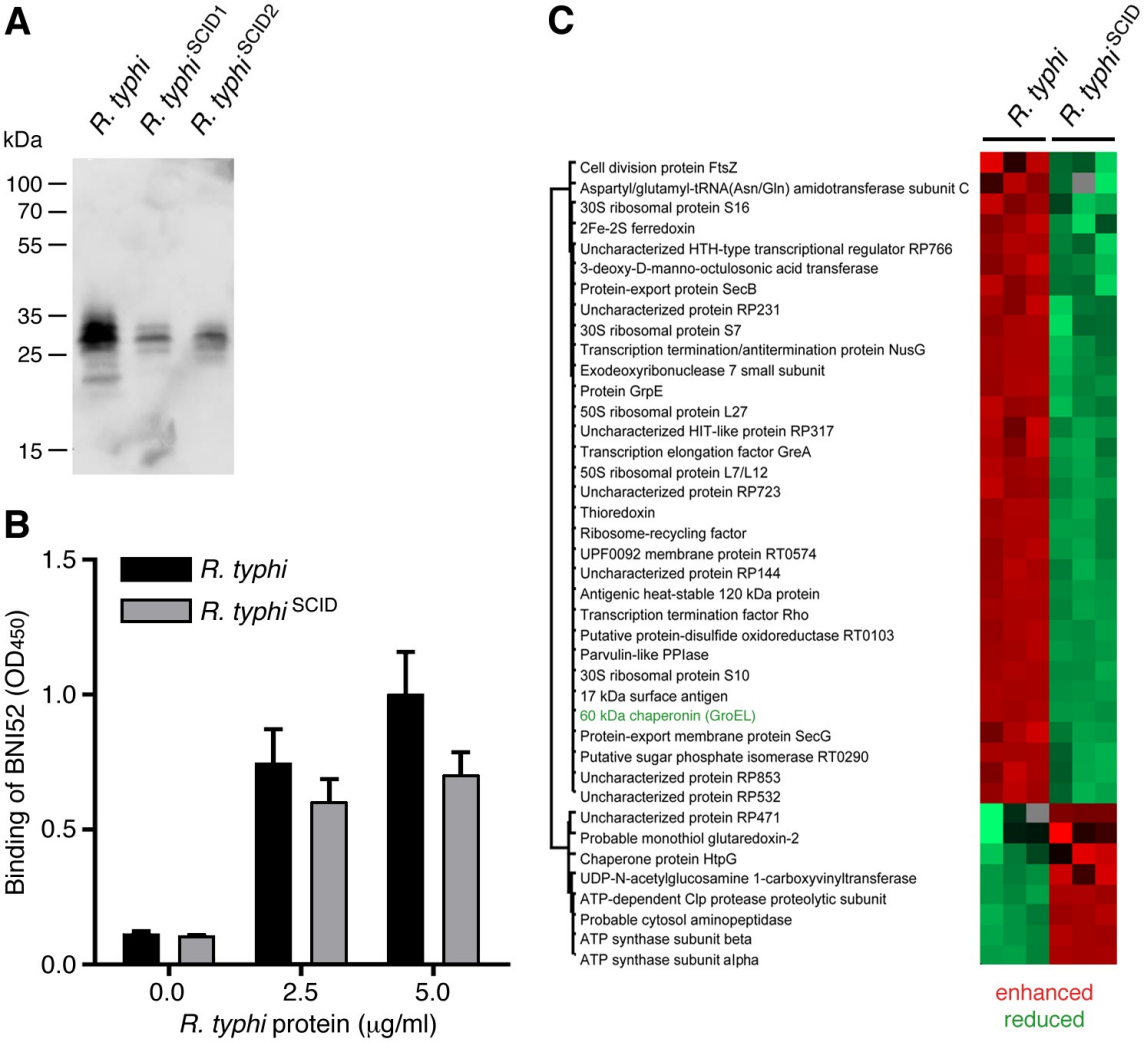
GroEL is downregulated by *R*. *typhi* after passage through BALB/c CB17 SCID mice. **(A)** *R*. *typhi* bacteria were reisolated from the spleens of BALB/c CB17 SCID mice and cultured in L929 cells *in vitro*. Bacteria reisolated from two different mice (*R*. *typhi*^SCID1^, *R*. *typhi*^SCID2^) were purified from L929 cell cultures and lysates were prepared for Western blotting in comparison to lysates from *R*. *typhi* prior to the passage through BALB/c CB17 SCID mice. 6 μg protein of each lysate was loaded. The membrane was incubated with BNI52. The antibody detects the same 25–35 kDa protein bands in *R*. *typhi*^SCID^ lysates as in *R*. *typhi* prior to the infection of the mice but with remarkably reduced signal intensity. **(B)** An ELISA with the indicated amounts of proteins in the lysate of *R*. *typhi* prior to the infection of BALB/c CB17 SCID mice (*R*. *typhi*) and re-isolated bacteria from these animals (*R*. *typhi*^SCID^) (x-axis) was performed. Plate-coated proteins were detected with the BNI52 antibody. The OD_450_ is depicted as a measure for the binding of BNI52 (y-axis). The binding of BNI52 to plate-coated *R*. *typhi* antigen from *R*. *typhi*^SCID^ was reduced compared to antigen from *R*. *typhi* prior to the infection of CB17 SCID mice. **(C)** Finally, the protein content in the lysates from *R*. *typhi* and *R*. *typhi*^SCID^ bacteria (reisolate from one mouse) was analyzed three times each and quantified by mass spectrometry. With this method differentially regulated proteins in *R*. *typhi* and *R*. *typhi*^SCID^ were identified (cut-off: ≥2fold change in expression). The expression of 32 proteins was reduced in *R*. *typhi*^SCID^ bacteria compared to *R*. *typhi* prior to the infection of CB17 SCID mice and the expression of 8 proteins was enhanced. The GroEL protein that is recognized by BNI52 was found among the proteins that are downregulated in *R*. *typhi*^SCID^ bacteria.

Further Western Blot studies (Fig 8A) and an ELISA (Fig 8B) were performed with lysates from *R*. *typhi* prior to the infection of BALB/c CB17 SCID mice and recovered *R*. *typhi*^SCID^. In both Western Blot and ELISA incubated with the BNI52 antibody the antigen was still detectable but its expression was strongly reduced in *R*. *typhi*^SCID^ compared to *R*. *typhi* prior to the passage through BALB/c CB17 SCID mice. To finally confirm the hypothesis that the expression of the antigen recognized by BNI52 is downregulated in *R*. *typhi* reisolates from BALB/c CB17 SCID mice, regulated proteins in *R*. *typhi*^SCID^ were examined by mass spectrometry in comparison to *R*. *typhi* prior to the passage through BALB/c CB17 SCID mice. These analyses revealed that 32 proteins were downregulated while the expression of 8 proteins was enhanced in *R*. *typhi*^SCID^ in comparison to *R*. *typhi* prior to the infection of CB17 SCID mice (Fig 8C). The GroEL protein was one of those proteins that were downregulated in *R*. *typhi* reisolates from BALB/c CB17 SCID mice. Thus, it seems that the absence of pressure through the adaptive immune system in BALB/c CB17 SCID mice influences the expression of a series of proteins including several immunoreactive ones such as FtsZ, PrsA, Sca4, 17 kDa surface antigen [43–47] and GroEL that is recognized by the BNI52 antibody.

## Discussion

In this study GroEL was identified as an immunodominant antigen of TG rickettsiae that is recognized by the BNI52 antibody as well as by antibodies in the sera from infected patients and immunized or infected mice. By electron microscopy and the precipitation of biotinylated surface proteins we further demonstrate that the ±30 kDa fragments that apparently derive from the GroEL protein and are recognized by BNI52 are surface-exposed and present in the periplasmic space but not in the cytosol of the bacteria. The BNI52 antibody clearly binds to recombinant *R*. *typhi* GroEL that was expressed and purified from *E*. *coli*. The epitope that is bound by this antibody must be linear because BNI52 detects the recombinant GroEL protein as well as the antigenic GroEL fragments in denatured *R*. *typhi* lysate samples in Western blots. The exact peptide sequence of the *R*. *typhi* GroEL protein that is recognized by the BNI52 antibody has not been determined, yet, but likely lies within the N-terminal part (amino acids 14–308) of the GroEL protein that is covered by the peptides identified by mass spectrometry (S1A Fig). Despite the strong homology of *R*. *typhi* GroEL with the corresponding protein from SFG rickettsiae [48], these GroEL fragments are specifically recognized by the BNI52 antibody on TG rickettsiae and bound by the antibody to a much higher extent than the full-length 60 kDa GroEL protein.

In line with the observations for TG rickettsiae, the GroEL protein has been shown to be an immunodominant antigen of SFG rickettsiae such as *R*. *conorii*, *R*. *helvetica*, *R*. *parkeri* and *R*. *heilongjiangensis* being recognized by antibodies in the sera from patients as well as animals infected with these pathogens [43, 45–47]. GroEL is predominantly expressed in the cytosol where it acts as a molecular chaperone together with the co-chaperone GroES and mediates the folding of previously unfolded or only partially folded substrate proteins [49]. This complex is involved in nearly all processes of protein biogenesis including the secretion of several proteins in Gram-negative bacteria [50]. In addition to the cytoplasm, GroEL was also found on cell surfaces or in membrane fractions of several bacteria including rickettsiae. Surface exposition of GroEL has been shown for *R*. *typhi* [51], *R*. *conorii* [43, 52], *R*. *rickettsii* [53], *R*. *heilongjiangensis* [46], *R*. *felis* [54] and *R*. *parkeri* [44] as well as several other pathogenic and non-pathogenic Gram-negative bacteria such as *Vibrio cholerae* [55], *Coxiella burnetii* [56], *Mycobacterium (M*.*) tuberculosis* [57], *M*. *smegmatis* [58], *M*. *avium* [59], *M*. *callisepticum* [60] and many others. In addition, GroEL has been shown to be secreted by several bacteria including *Francisella tularensis* growing intracellularily in macrophages [61], *Helicobacter pylori* [62], *Bacillus* (*B*.) *subtilis* [63] and *B*. *anthracis* [64] where the protein is also found on the bacterial surface [65]. Whether GroEL is secreted by *R*. *typhi* as well remains to be investigated.

How GroEL is secreted or gets access and attached to the bacterial cell surface is unknown. Exported proteins have to pass the periplasm to be integrated into the outer membrane. In our electron microscopy studies the antigen that is bound by BNI52 was indeed found in the periplasmic space. As a chaperone that is predominantly required in the cytosol, GroEL does not contain a classical signal sequence. GroEL, however, has been shown to actively participate in the Sec and twin-arginine translocation (Tat) pathways for the postranslational secretion of proteins [66]. One target of GroEL is the ATPase SecA [67], a central cytosolic component of the Sec translocation system that delivers substrate proteins to the Sec translocon and drives the ATP-dependent translocation [68]. Furthermore, GroEL has been shown to interact with outer membrane proteins such as OmpA as well as with periplasmic proteins [69] some of which are SecB substrates [70, 71]. These translocation systems in addition to others (T1SS, T4SS, T5SS) are expressed by rickettsiae [72]. A possible mechanism of surface exposition of GroEL might therefore be its interaction either with proteins of the translocation machineries or exported proteins or both. In the protein spots detected by BNI52, OmpB was the only protein apart from GroEL from which peptides were detected by mass spectrometry. These peptides almost exclusively derived from the p32 peptide of the OmpB protein that is separated from OmpB during maturation and export. However, neither recombinantly expressed p32 peptide nor fragments of mature OmpB were recognized by BNI52, excluding OmpB as a target for this antibody. These results rather suggest that GroEL interacts with OmpB and assists in the folding, maturation and export of this protein. It is imaginable that GroEL binds to the p32 peptide from OmpB which is then co-exported with the surface-exposed fragment of GroEL during this process, and that the BNI52 antibody co-precipitates the p32 peptides together with GroEL.

It is surprising, however, that the BNI52 antibody does not detect its antigen in the cytosol of the bacteria in our electron microscopy studies. An explanation for that could be that the linear antigenic epitope is sterically blocked in the folded intact cytosolic GroEL protein but freely accessible in the periplasmic and surface-exposed fragments. This, however, cannot explain why BNI52 detects the denatured full length GroEL from *R*. *typhi* only weakly in Western Blots while recombinant GroEL from *E*. *coli* is clearly recognized. One possibility is that the cytosolic GroEL protein in rickettsiae carries posttranslational modifications that are missing in the recombinant protein from *E*. *coli* and mask the antigenic epitope. These may also not be present on the surface-exposed ±30 kDa protein fragments that are recognized by the BNI52 antibody much stronger.

The fragments can result from proteolytic degradation of the GroEL protein that may occur in the periplasm. The periplasm is a multipurpose cellular compartment that provides an oxidizing environment as well as several enzymes including proteases, glycosyltransferases and kinases that can modify exported or outer membrane proteins by many different ways [73]. Proteolytic cleavage of proteins may enhance the functional diversity [74], especially in organisms with a limited repertoire of proteins as rickettsiae that have very small genomes (1.1–1.3 Mb). The peptides that were identified by mass spectrometry in all ±30 kDa protein spots that were recognized by BNI52 derived from the region 14–308, suggesting that there is at least cleavage of the protein into two parts. Accidental cleavage into so many fragments that appear as a kind of graduated protein ladder (25, 27, 30, 32, 35 kDa), all of them recognized by BNI52, however, seems to be unlikely.

A more probable explanation for that is that a single fragment of the GroEL protein is generated that carries posttranslational modifications. Similar protein ladders, for example, are commonly observed for eukaryotic glycosylated proteins where different amounts of sugars of defined molecular weight are attached to the protein. It is now well accepted that *O*- as well as *N*-linked glycosylation of proteins occur in prokaryotes as well [75] including members of the order *Rickettsiales*. For example, it has been shown that the immunodominant proteins P120 and P140 of *Ehrlichia* (*E*.*) chaffeensis* and *E*. *canis* are glycosylated [76]. This is also true for the GroEL proteins from these bacteria [77] as well as the GroEL protein from *E*. *ruminantium* that was found to be *O*-GlcNAcylated [78]. According to the Glycopp prediction server (https://webs.iiitd.edu.in/raghava/glycopp/ [79]), four *O*- and 5 *N*-glycosylation sites exist in the *R*. *typhi* GroEL protein sequence 14–308 that is covered by the peptides identified by mass spectrometry (S1B Fig). In addition, the GroEL fragments that are predominantly detected by BNI52 appeared with a very low pI of about 3 while full length GroEL protein was found within a pI range from 5–7. This observation argues for a strong acidification of the protein fragments. This could be due to phosphorylation that adds negative charge and turns a protein acidic. The GroEL proteins from *Ehrlichia* species have been demonstrated to carry phosphorylations in addition to glycosylations as well as lipoylations [77]. Also, phosphorylation of GroEL from *M*. *tuberculosis* has been described [80]. The GroEL protein from *B*. *anthracis* has been shown to contain six Thr sites that are phosphorylated by the protein kinase C (PrkC). Here, the protein is found in four isoforms of which three have a low pI near 4 [81], similar to the GroEL fragments that are recognized by the BNI52 antibody. Such posttranslational modifications can affect signaling events, regulatory processes, protein stability, molecular interactions and localizations. Especially protein phosphorylation has been associated with the regulation of housekeeping processes and virulence [82]. In support of that it was found that the phoshorylation of *B*. *anthracis* GroEL by the PrkC kinase leads to activation of the protein which is important for biofilm formation [81]. Similarly, the phosphorylation of GroEL from *M*. *tuberculosis* has been shown to facilitate the oligomerization of the protein and to enhance its function in this way [80]. Five potential phosphorylation sites, exclusively Ser, are present in the GroEL sequence 14–308 from *R*. *typhi* (S1B Fig) according to MPSite, a predictor for microbial phosphorylation sites (http://kurata14.bio.kyutech.ac.jp/MPSite [83]). Glycosylations as well as phosphorylations can be possibly also acquired in the periplasm where glycosyltransferases and protein kinases are present [74]. However, it is unknown whether rickettsiae possess the enzymes that are capable to recognize and phosphorylate or glycosylate the mentioned sites. In this context it is interesting that several phosphorylated proteins were found in *E*. *rumantium* although corresponding enzymes that could be responsible for these phosphorylations were not detectable in the bacterial proteome [78]. Therefore, it may be finally discussed whether surface-exposed molecules of intracellular bacteria are accessible for eukaryotic cytosolic enzymes that could add protein modifications. For *Chlamydia* sp. it has been shown that these bacteria indeed use host cell kinases for the phosphorylation of T3SS effector proteins that are involved in cell invasion [84]. In addition to the determination of the antigenic epitope that is recognized by BNI52, it will be interesting to elucidate the presence and the nature of possible posttranslational modifications of the GroEL protein from TG rickettsiae. These future analyses will show whether the epitope differs from the corresponding peptide in the strongly homologues protein from SFG rickettsiae that is obviously not recognized by BNI52, and whether GroEL from TG rickettsiae carries posttranslational modifications that may be unique for these bacteria. Such modifications can also contribute to immune reactivity.

Many surface-exposed proteins have important functions for the interaction of bacteria with the environment and with host cells. This is especially true for the members of the surface cell antigen (Sca) family of proteins, especially Sca0 (OmpA) and Sca5 (OmpB) [85–90]. OmpA and OmpB are involved in bacterial adhesion to host cells [91, 92]. Within the Sca family, Sca4 is not presented on the cell surface of the bacteria but expressed in the cytosol [93] and also found to be secreted from *R*. *typhi* [87] and *R*. *parkeri* [94] where it has been shown to interact with the cell adhesion protein vinculin [87, 94] and to be critical for bacterial cell-to-cell spread for *R*. *parkeri* [94]. Interestingly, surface-exposed GroEL of *M*. *tuberculosis* has been shown to be important for the attachment and uptake of the bacteria by macrophages [95]. It was further demonstrated that GroEL is a ligand of the sialylated surface glycoprotein CD43 which mediates adhesion and phagocytosis of *M*. *tuberculosis* by macrophages [96]. Involvement of GroEL on the surface of rickettsiae in the attachment and uptake of the bacteria is therefore imaginable. In addition, surface proteins are important antigens that activate innate and adaptive immune cells and induce protective immune responses against rickettsial infections, especially the production of antibodies for which these antigens are accessible. The two major outer membrane proteins OmpA and OmpB of SFG rickettsiae elicit humoral and T cell-mediated immune responses, and immunization with recombinant OmpA or OmpB of *R*. *rickettsii* or *R*. *conorii* protects guinea pigs and mice against infection with these pathogens [97–99]. Similarly, treatment with purified native OmpB of *R*. *typhi* provides complete protection in mice challenged with *R*. *typhi* [100, 101]. Specific antibodies are produced rather late in the infection with rickettsiae and are considered to play a minor role in defense in primary infection. Nonetheless, they can contribute to protection in secondary infection or upon immunization by different mechanisms such as opsonization, complement activation or the inhibition of cellular invasion. Antibodies against OmpA have been shown to inhibit adherence of *R*. *rickettsii* to L929 cells *in vitro* [86], and passive immunization with polyclonal anti-*R*. *conorii* serum or monoclonal antibodies against OmpA or OmpB even protected immunodeficient C3H/HeN SCID mice against challenge with this pathogen [102, 103]. It was observed that BNI52 opsonizes the bacteria for the uptake by phagocytic cells, especially macrophages. Whether the opsonizing effect of BNI52 can contribute to protection *in vivo* remains to be investigated, but the observation indicates that it is mediated by receptors that are present on specialized phagocytic immune cells.

Employing mass spectrometry we found that the BNI52 antibody precipitates fragments of the GroEL protein. Furthermore, it does not recognize its antigen anymore after digestion of *R*. *typhi* proteins with proteinase K. Finally, the BNI52 antibody binds to recombinantly expressed GroEL. These findings clearly show that the antibody recognizes the GroEL protein rather than LPS or other polysaccharide cell wall components.

It is also interesting that the GroEL protein was found to be one of 40 proteins that are obviously differently regulated in *R*. *typhi*. Differential regulation of proteins has also been demonstrated for *R*. *prowazekii*, the closest relative of *R*. *typhi*, when grown in murine, tick, and insect cell lines or in egg yolk sacs [104]. Moreover, GroEL has been shown to be upregulated by *R*. *prowazekii* in the early phase of infection during cellular invasion (10–30 min) [105], maybe indicating an enhanced need for its chaperone activity in this situation. Enhanced expression of GroEL is also observed in *R*. *prowazekii* when infecting human microvascular endothelial cells (HMEC) compared to *Amblyomma americanum* AAE2 arthropod cells *in vitro* [106]. This regulation may be a result of the temperature of culture (HMEC are grown at 37°C, AAE2 cells at 34°C) but could be also dependent on the nature of the cells. In contrast to that, in *R*. *typhi*^SCID^ bacteria that were reisolated from BALB/c CB17 SCID mice the GroEL protein was found among those 32 proteins that were downregulated compared to *R*. *typhi* prior to the passage through these animals.

The majority (thirteen proteins) of the downregulated proteins are involved in transcription, ribosome assembly and translation. These include the transcriptional activator HTH-type transcriptional regulator (RP765), the transcription termination/antitermination protein NusG (RT0124), the exoribonuclease 7 small subunit (RT0619), the transcription termination factor Rho (RT0513), the transcription elongation factor GreA (RT0850) which is necessary of efficient RNA polymerase transcription elongation, the 30S ribosomal proteins S10 (RT0652), S16 (RT0869) and S7 (RT0120) and the 50S ribosomal proteins L7/L12 (RT0128) and L27 (RT0737), the ribosome recycling factor (RT0143) and the aspartyl/glutamyl-tRNA (Asn/Gln) amidotransferase subunit C (GatC, RT0142). One downregulated protein, HIT-like protein (RP317), might be involved in gene regulation by binding to small nucleolar ribonucleic acids (snoRNAs) [107]. Five of the downregulated proteins are housekeeping proteins involved in the iron-sulfur cluster biogenesis (2Fe2S ferredoxin, RT0189), a putative protein-disulfide oxidoreductase (RT0103) involved in redox homeostasis [108], and a putative sugar phosphate isomerase (RT0290) which is part of the carbohydrate metabolic process. Apart from GroEL three other proteins involved in stress response, cell cycle, protein secretion and cell division were downregulated. These include SecB (RT0062), GrpE (RT0620), a cofactor of the bacterial Hsp70 protein DnaK, thioredoxin (RT0002), a protein that is involved in the oxidative stress response and that has multiple cellular functions including gene regulation [109], and FtsZ (Filamenting temperature-sensitive mutant Z, RT0658), the homologue of the eukaryotic tubulin which is essential for cell division [110]. The 3-deoxy-D-manno-octulosonic acid transferase (Kdo transferase, RT0048) which is involved in the generation of the lipid A component of LPS [111] was downregulated in *R*. *typhi*^SCID^ as were the surface proteins 17 kDa surface antigen (RT0821) and PrsA (parvulin-like PPIase, RT0565) as well as the cytosolic and exported antigenic Sca4 protein (RT0485). The remaining downregulated proteins are not further characterized (RP144, RP231, RP723, RP853 and RP532). Of these the latter three are predicted to be integral membrane proteins.

Interestingly, several of the downregulated proteins have immunological properties and either act immunomodulatory (GroEL) and/or are immunogenic antigens themselves (GroEL, FtsZ, PrsA, Sca4, 17 kDa surface antigen [43–47]. The reason for the downregulation of these proteins is not clear, but may be a result of the absence of pressure through the adaptive immune system in BALB/c CB17 SCID mice. These regulated proteins may therefore play a role in virulence and/or pathogenicity.

## Concluding remarks

Taken together, this is the first report demonstrating that GroEL is an immunodominant antigen of TG rickettsiae. So far, it has been described that antibodies against this protein are raised in the infection with SFG rickettsiae, e.g. *R*. *conorii* [43] and *R*. *heilongjiangensis* [46], and it has been shown that GroEL is the predominant antigenic protein in *E*. *muris* and *E*. *chaffeensis* [77]. For several bacterial infections GroEL has been shown to have a great potential for the use as a vaccine. Immunization of mice with *B*. *anthracis* GroEL leads to a humoral as well as to a robust T cell response and protects mice against the infection [112]. Similarly, protective immunity upon immunization with GroEL has been demonstrated for the infection with *M*. *tuberculosis*, *Helicobacter pylori*, *Salmonella typhimurium*, and *Streptococcus pneumoniae* [61, 113–118]. Future investigations will show whether the rickettsial GroEL protein also has the potential as a vaccine candidate. Apart from that, the BNI52 antibody represents a tool for the specific detection of TG rickettsiae in various experimental and diagnostic settings such as Western blotting, immunofluorescence stainings and electron microscopy. In addition, the antibody has been successfully applied for flow cytometric and immunohistological detection of *R*. *typhi* in infected mice [12, 42].

## Supporting information

S1 Fig Predicted phosphorylation and glycosylation sites in the GroEL protein from *R*. *typhi*. **(A)** The sequence of the *R*. *typhi* GroEL protein is depicted. The peptide sequences identified by mass spectrometry in the ±30 kDa spots detected by BNI52 cover the region 14–308 (gray background) and are labeld by dark gray background. **(B)** The protein sequence of GroEL was analyzed for the presence of phosphorylation and glycosylation sites employing MPSite and Glycopp prediction algorithms (BPP: Prediction based on Binary Profile Patterns; CPP: Prediction based on Composition Profile Patterns; PPP: Prediction based on PSSM Profile patterns; ASA+PPP: Prediction based on Average Surface Accessibility). The prediction scores are given. High probability scores are highlighted in green. The predicted phosphorylation and glysolytation sites are colored in the protein sequence in (A) according to the background coloring of these sites in the table.
(TIF)

S2 Fig The BNI52 antibody does not bind to the p32 peptide of OmpB nor to mature OmpB. **(A)** The OmpB protein sequence from *R*. *typhi* is shown. The p32 peptide that is eliminated from the protein during the maturation and export process is labeled in light grey. The peptides that were found by the MS analyses of BNI52 precipitates are highlighted in dark grey. Except for one peptide all of them derived from the p32 peptide of OmpB. His-tagged GroEL, p32 peptide and overlapping fragments of the mature OmpB protein (OmpBF1, OmpBF2, OmpBF3) of *R*. *typhi* were expressed in *E*. *coli* (GroEL and p32 peptide) or in HEK293T cells (fragments of mature OmpB). Purified GroEL (1 μg), p32 peptide (1 and 3 μg) and lysates of HEK293T cells expressing the fragments of mature OmpB were applied to SDS Page and Western blotting. In case of the latter, lysates from non-transfected cells (untreated) or cells transfected with empty control vector (control) were used as a control. A lysate of *R*. *typhi* bacteria was included as an additional control. The membranes were incubated with a polyhistidine antibody or BNI52 as indicated below. All recombinant proteins were detectable with the anti-His antibody while the BNI52 antibody neither bound to the p32 peptide nor fragments of mature OmpB.
(TIF)

S3 Fig BNI52 does not detect *R*. *typhi*^SCID^ in L929 *in vitro* cell cultures. L929 cells were infected with *R*. *typhi*^SCID^ (left) and *R*. *typhi* prior the passage through BALB/c CB17 SCID mice (right) and stained with the BNI52 antibody (green). Nuclei were stained with DAPI (blue). *R*. *typhi*^SCID^ are not detectable anymore with the BNI52 antibody by this method.
(TIF)

S1 Raw images (PDF)

## Abbreviations

bm: bone marrow-derived
BNI52: anti-*R*. *typhi* antibody BNI52 (IgG3 combined with a λ light chain)
DC: dendritic cell
MΦ: macrophage
*R*.: rickettsia
SFG: spotted fever group
TG: typhus group
