# Supplementary figures and images for: GroEL is an immunodominant surface-exposed antigen of Rickettsia typhi
Source: PLoS One. 2021 Jun 10;16(6):e0253084. doi: 10.1371/journal.pone.0253084 (PMC8191997; doi:10.1371/journal.pone.0253084)

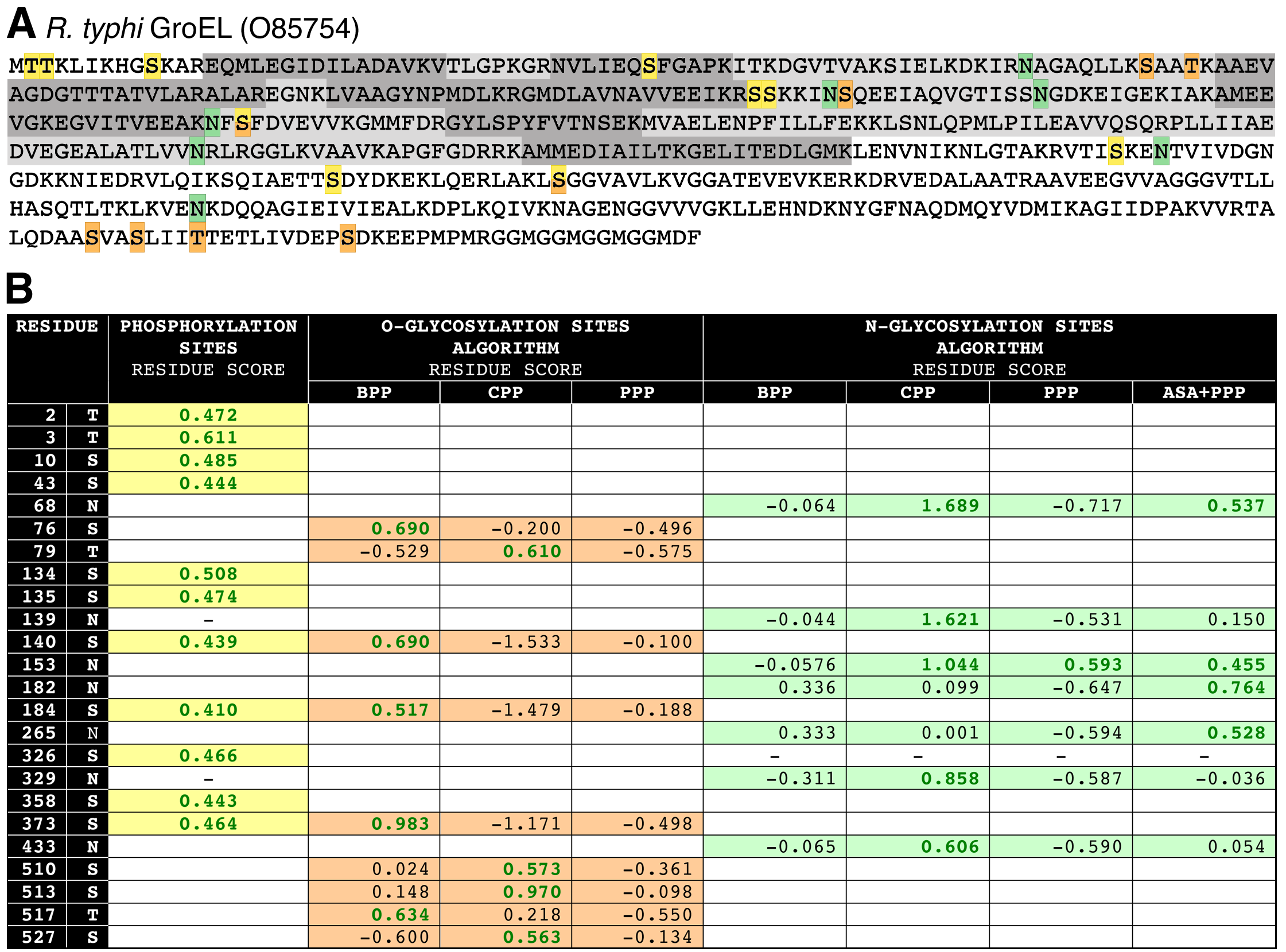

Supplement: S1 Fig — (A) The sequence of the R. typhi GroEL protein is depicted. The peptide sequences identified by mass spectrometry in the ±30 kDa spots detected by BNI52 cover the region 14–308 (gray background) and are labeld by dark gray background. (B) The protein sequence of GroEL was analyzed for the presence of phosphorylation and glycosylation sites employing MPSite and Glycopp prediction algorithms (BPP: Prediction based on Binary Profile Patterns; CPP: Prediction based on Composition Profile Patterns; PPP: Prediction based on PSSM Profile patterns; ASA+PPP: Prediction based on Average Surface Accessibility). The prediction scores are given. High probability scores are highlighted in green. The predicted phosphorylation and glysolytation sites are colored in the protein sequence in (A) according to the background coloring of these sites in the table. (TIF) [file pone.0253084.s001.tif]

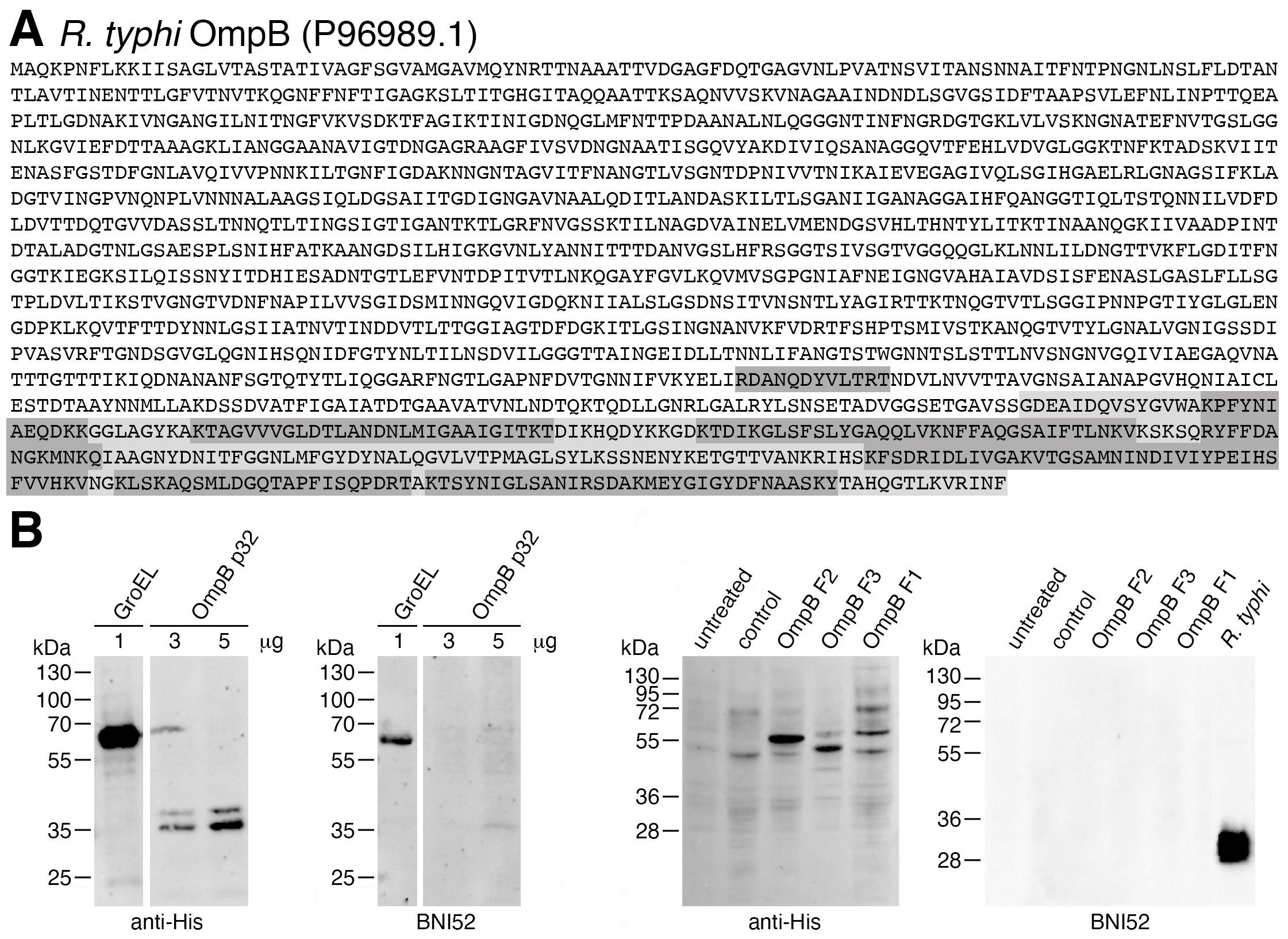

Supplement: S2 Fig — (A) The OmpB protein sequence from R. typhi is shown. The p32 peptide that is eliminated from the protein during the maturation and export process is labeled in light grey. The peptides that were found by the MS analyses of BNI52 precipitates are highlighted in dark grey. Except for one peptide all of them derived from the p32 peptide of OmpB. His-tagged GroEL, p32 peptide and overlapping fragments of the mature OmpB protein (OmpBF1, OmpBF2, OmpBF3) of R. typhi were expressed in E. coli (GroEL and p32 peptide) or in HEK293T cells (fragments of mature OmpB). Purified GroEL (1 μg), p32 peptide (1 and 3 μg) and lysates of HEK293T cells expressing the fragments of mature OmpB were applied to SDS Page and Western blotting. In case of the latter, lysates from non-transfected cells (untreated) or cells transfected with empty control vector (control) were used as a control. A lysate of R. typhi bacteria was included as an additional control. The membranes were incubated with a polyhistidine antibody or BNI52 as indicated below. All recombinant proteins were detectable with the anti-His antibody while the BNI52 antibody neither bound to the p32 peptide nor fragments of mature OmpB. (TIF) [file pone.0253084.s002.tif]

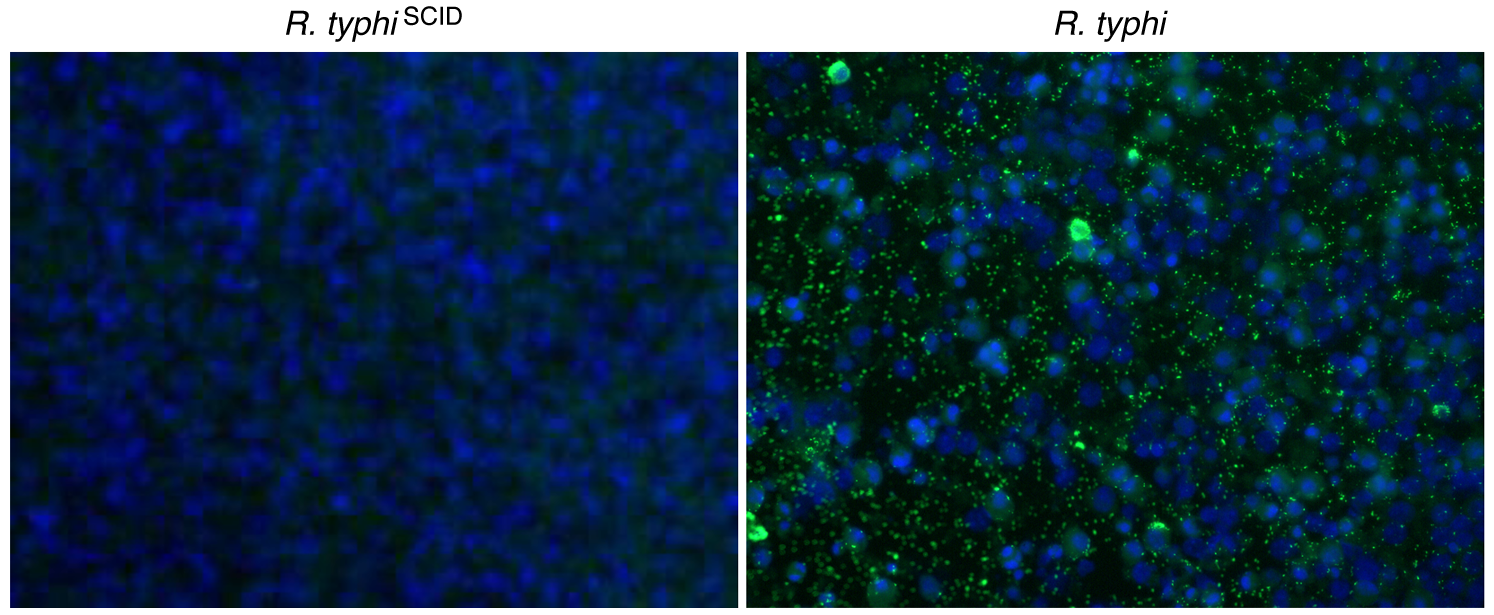

Supplement: S3 Fig — L929 cells were infected with R. typhiSCID (left) and R. typhi prior the passage through BALB/c CB17 SCID mice (right) and stained with the BNI52 antibody (green). Nuclei were stained with DAPI (blue). R. typhiSCID are not detectable anymore with the BNI52 antibody by this method. (TIF) [file pone.0253084.s003.tif]
