# Supplementary material for: GroEL is an immunodominant surface-exposed antigen of Rickettsia typhi
Source: PLoS One. 2021 Jun 10;16(6):e0253084. doi: 10.1371/journal.pone.0253084 (PMC8191997; doi:10.1371/journal.pone.0253084)

Original Image of Fig 3A.

Proteins in the lysate of *R. typhi* were digested with Proteinase K for indicated periods. Control bacterial lysate was left undigested. A Western Blot was performed. The membrane was incubated with the BNI52 antibody followed by the incubation with a HRP-labeled secondary antibody.

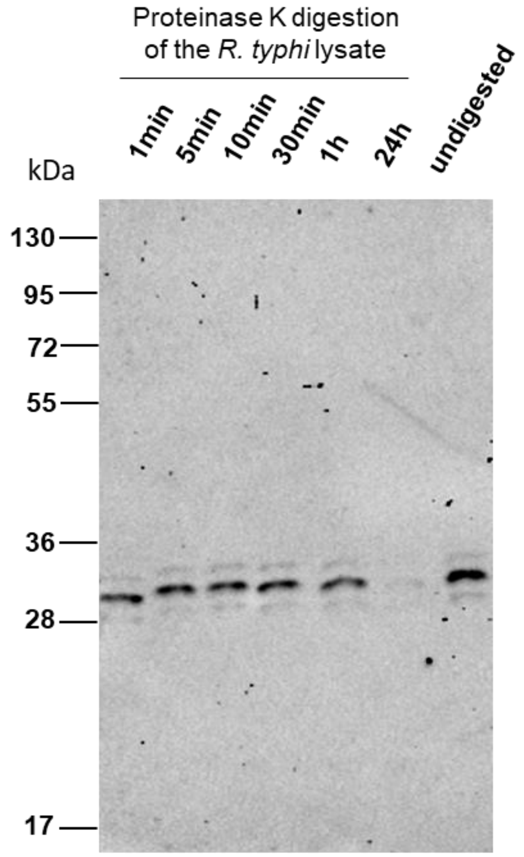

Original Images of Fig S2B.

Purified GroEL (1 µg), OmpB p32 peptide (1 and 3 µg) were applied to SDS Page and Western blotting. The membranes were incubated with BNI52 (left) or a polyhistidine antibody (right).

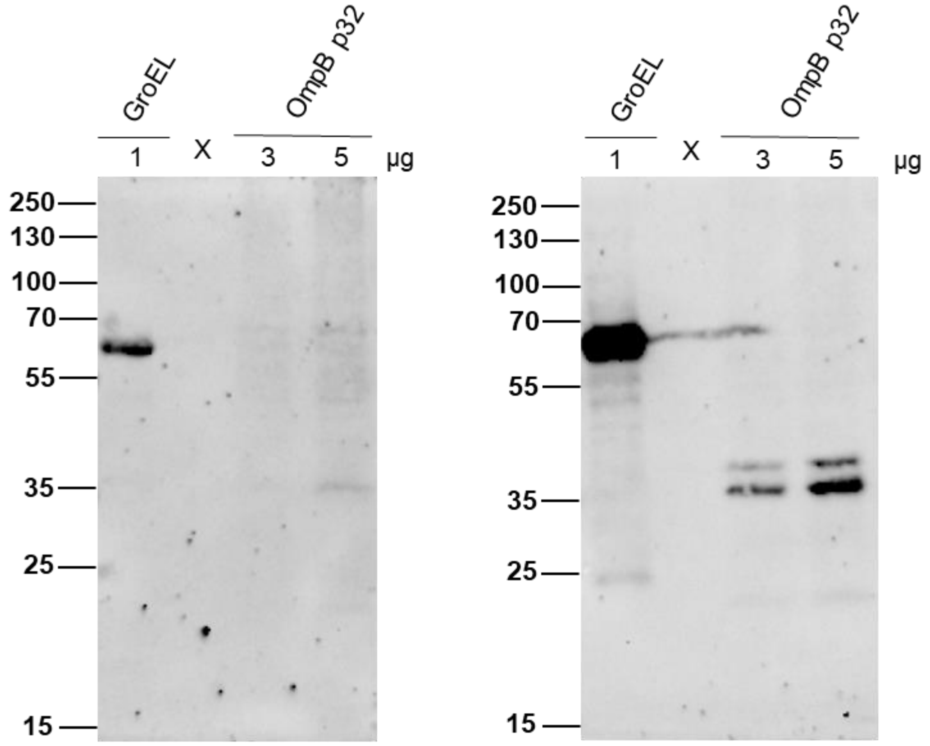

Supplement: S1 Raw images — (PDF) [file pone.0253084.s004.pdf]
